# Methane dynamics in gas chimneys linking geochemical and microbial methane cycling in the Ulleung basin

**DOI:** 10.1186/s12866-025-04547-7

**Published:** 2025-11-22

**Authors:** Dukki Han, Jiyoung Choi, Kwangchul Jang, Bo-Yeon Yi, Ji-Hoon Kim

**Affiliations:** 1 Department of Marine Molecular Bioscience, Gangneung-Wonju National University, Gangneung, 25457 Republic of Korea; 2 Resource Exploration and Development Research Division, Korea Institute of Geoscience and Mineral Resources, Daejeon, 34312 Republic of Korea; 3 Department of Earth System Sciences, Yonsei University, Seoul, 03722 Republic of Korea

**Keywords:** Gas chimney, SMTZ, Ulleung basin, Metabarcoding, Microbial communities

## Abstract

**Background:**

Gas chimneys in marine sediments act as conduits for methane (CH_4_) migration and influence geochemical and microbial processes. The sulfate–methane transition zone (SMTZ), where CH_4_ is oxidized and sulfate (SO_4_^2−^) is reduced, plays a key role in carbon cycling. Temperate continental margins, such as the Ulleung Basin in the East Sea of Korea, are particularly sensitive to environmental changes that may influence methane flux and hydrate stability. We analyzed sediment cores from chimney (Sites P03, P04) and non-chimney (Site P01) structures to examine how CH_4_ flux and hydrate dynamics shape SMTZ depth and microbial communities.

**Results:**

Geochemical analyses showed that the SMTZ was located deeper at the non-chimney site (P01), with stable salinity and isotopic composition, and no evidence of hydrate dissociation. In contrast, chimney sites (P03 and P04) had shallower SMTZs. Site P03 exhibited reduced salinity and chloride concentrations and enriched δD and δ^18^O values, indicating hydrate dissociation. SO_4_^2−^ and CH_4_ profiles further defined SMTZ depth, where their redox processes were spatially coupled. Carbon isotopic profiles from porewater and gas indicated active anaerobic methane oxidation near the SMTZ. In hydrate-bearing sediments, particularly at Site P03, microbial communities were dominated by JS1 and *Lokiarchaeia*, which are associated with CH_4_ oxidation and SO_4_^2−^ reduction. Functional predictions, consistent with geochemical trends, showed SO_4_^2−^ reduction as the dominant microbial process in the SMTZ, while methanogenesis peaked at the zone and declined below. Chimney-specific taxa, including carboxydotrophic and fermentative bacteria, were also enriched at Site P03, suggesting adaptation to elevated CH_4_ flux and diverse carbon sources.

**Conclusion:**

This study enhances our understanding of carbon cycling in temperate marine sediments, where hydrate destabilization can significantly influence methane fate and microbial community responses.

**Supplementary Information:**

The online version contains supplementary material available at 10.1186/s12866-025-04547-7.

## Background

In deep subseafloor sediments with high organic carbon deposits, microorganisms decompose organic matter to produce methane (CH_4_) [1], which migrates upward through the sediments. However, methane becomes trapped within a lattice of hydrogen-bonded frozen water molecules under high-pressure and low-temperature conditions along continental margins, forming gas hydrates [2]. Gas chimney structures are often associated with gas hydrate near the seafloor and act as major conduits for gas migration from deeper reservoirs to the seafloor [3, 4]. These features form when gas generated through biogenic or thermogenic processes accumulates under pressure and migrates through structural weaknesses in the sediment, such as fractures or faults [3]. Hydrate-bearing sediments play a crucial role in the marine ecosystem by influencing sediment stability as well as geochemical and microbial dynamics. For example, hydrate structures impart rigidity and stability to sediments [5]; however, their dissociation due to changes in temperature or pressure causes sediment destabilization. In addition to these physical effects, hydrate-bearing sediments significantly impact marine geochemistry and microbial ecology by altering the distribution of CH_4_ and sulfate (SO_4_^2−^), giving rise to distinct zones such as the sulfate–methane transition zone (SMTZ) [2].

The SMTZ is a central hub in the subseafloor carbon cycling process, in which CH_4_ is primarily consumed through anaerobic oxidation of methane (AOM) and converted into CO_2_. This process acts as a natural filter that effectively prevents CH_4_ emission from marine sediments into the atmosphere. This zone is characterized by a sharp decrease in SO_4_^2−^ concentration and a concurrent increase in CH_4_ concentration. Its position is dictated by the upward migration of CH_4_ and the downward diffusion of SO_4_^2−^. Chimney structures typically possess shallow SMTZs and intense CH_4_ fluxes that highlight the localized carbon cycling processes in the sediments. Moreover, these chimneys provide valuable insights into global CH_4_ emissions. The microbial communities in these regions are essential for the biogeochemical processes that occur in the SMTZ. Sulfate-reducing bacteria (SRB) and anaerobic methanotrophic archaea (ANME) act as key drivers of AOM by coupling CH_4_ oxidation with SO_4_^2−^ reduction [1], which indirectly influences gas hydrate stability and distribution by altering the surrounding geochemical conditions [2]. Therefore, the SMTZ is a dynamic interface between geological, geochemical, and microbial processes that regulate carbon cycling and CH_4_ flux and represents a critical component of both local and global biogeochemical cycles.

Hydrate-bearing sediments host diverse and complex microbial communities that are linked to biogeochemical processes such as carbon cycling (CH_4_ production and oxidation) and SO_4_^2−^ reduction [6]. Traditional methods such as cultivation-based approaches or molecular techniques targeting specific microbes are often ineffective in identifying unculturable or low-abundance taxa owing to methodological limitations [7–9]. In contrast, rRNA gene metabarcoding, which analyzes sedimentary DNA, offers a high-resolution approach to characterize microbial communities in diverse subseafloor environments [9–12]. This method aids in determining microbial biodiversity and functional potential under the environmental factors that influence hydrate stability.

The Ulleung Basin, located in the southwestern East Sea off the Korean Peninsula, is characterized by high biological productivity and abundant acoustic chimneys (see details in Supplementary information) that provide ideal conditions to investigate the interplay between CH_4_ flux, hydrate stability, and microbial community dynamics. Hydrate-bearing sediments such as gas chimneys in the Ulleung Basin are critical to understanding carbon cycling in the subseafloor. Consequently, the biogeochemical and geological factors that affect hydrate formation, stability, methane dynamics, and microbial ecology in this region have been extensively studied [13–23]. Nevertheless, critical aspects of the role of microbial communities in CH_4_ dynamics and their mediation of sulfur cycling in gas chimneys remain unclear. Moreover, high-throughput metabarcoding is yet to be applied in studying the Ulleung Basin despite its proven potential for aiding in the identification of microbial processes in marine sediments [24–26].

The aim of this study was to investigate the role of CH_4_-based geochemical zones in shaping microbial ecosystems and to examine the influence of gas hydrate proximity on microbial community organization. To address these aims, we integrated high-throughput metabarcoding with biogeochemical and geological analyses of the Ulleung Basin, focusing on differences in microbial communities across geochemical zones in CH_4_-rich marine sediments and the impact of gas hydrate proximity on community composition. Therefore, this study provides a comprehensive understanding of microbial ecology in hydrate-bearing sediments and its role in CH_4_ dynamics and carbon cycling.

## Methods

### Sample preparation

**Fig. 1:**
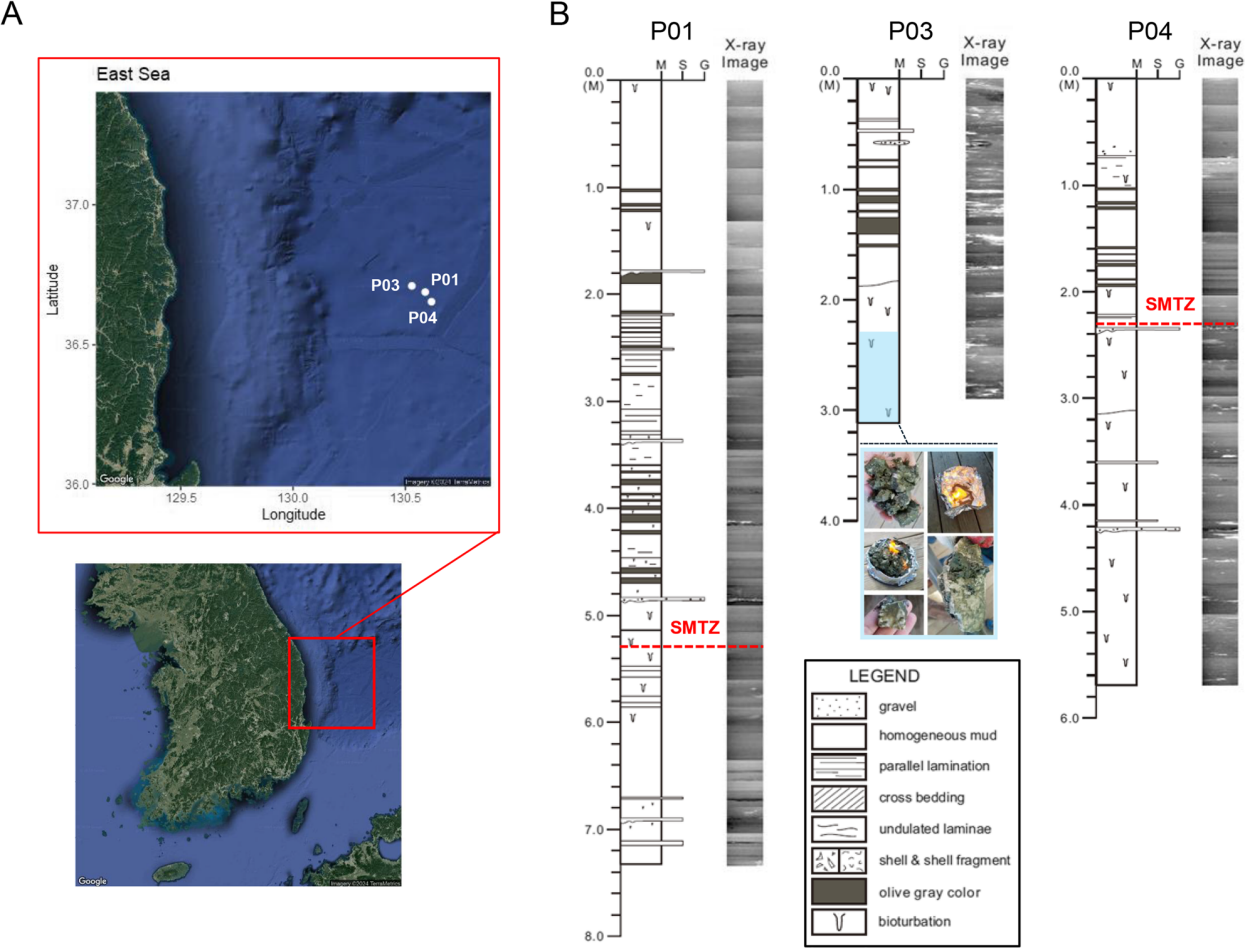
Description of sampled areas in the East Sea of Korea and environmental heterogeneity. **A** Locations of three coring sites (P01: non-chimney, P03 and P04: chimney structures), with coordinates provided in decimal degrees. **B** Geological description of sediment cores from the three sites. Core depths are shown in meters below seafloor (mbsf). The core descriptions are based on direct visual observations during core logging, documenting lithological structures, sedimentary layering, and textural variations. In addition, X-ray radiographs of the same cores were included to highlight density contrasts and internal sedimentary features. Red dashed lines indicate the depth of the sulfate–methane transition zone (SMTZ) at each site (P01 = 5.3 mbsf; P03 < 0.5 mbsf; P04 = 2.3 mbsf). The SMTZ at P03 is inferred to occur near the surface based on sulfate–methane trends and hydrate occurrence. The blue-shaded interval in P03 represents the hydrate-bearing zone observed in sediments below ~ 2.3 mbsf

In 2017, three sediment cores were collected from the southern Ulleung Basin during the 07GH Expedition of the R/V *Tamhae II* (Fig. 1A). Each core was subsampled at multiple depth intervals, and each depth-specific sample was treated as an independent unit for analysis. Due to logistical constraints, no additional replicate cores or technical replicates were obtained. Depth intervals varied among cores depending on recovery conditions, with priority given to capturing the SMTZ and hydrate-bearing layers. Two gas chimney structures at Sites 17GH-P03 (hereafter P03) and 17GH-P04 (hereafter P04) and one non-chimney structure at Site 17GH-P01 (hereafter P01) were targeted (Table S1). Although a core was retrieved from a chimney-like structure at Site 17GH-P02, no visible hydrate signal was observed and no comparable analytical data were obtained for this study. Geological descriptions of lithological structures, sedimentary layering, and textural variations were obtained to provide context for subsequent geochemical and microbial analyses (Fig. 1B). Gas hydrates were recovered onboard from Site P03, and their contaminated surfaces were carefully removed to ensure clean sampling for the analysis of dissociated hydrate gas and fluid. Gas samples were collected from sediment (headspace gas) and dissociated gas hydrates. Upon retrieval, the headspace gas samples were collected immediately after core cutting and sealed in 21-mL serum vials containing 2 mL of saturated NaCl solution. Gases from dissociated hydrates were also collected. All gas samples were stored at 4 °C until needed for further analysis.

Porewater was extracted onboard using Rhizons (Rhizosphere Research, Netherlands; diameter, 2.5 mm; length of porous media, 5 cm; pore size, 0.12–0.18 μm) at 30–130-cm intervals, depending on the core length. All samples were handled under cold storage conditions (approximately 4 °C) to preserve their integrity. Extracted porewater samples (< 20 mL) were filtered through a 0.2-µm polytetrafluoroethylene syringe filter. Subsamples for salinity, chloride (Cl^−^), alkalinity, nutrients (NH_4_^+^ and PO_4_ ^− 3^), SO_4_^2−^, and hydrogen sulfide (HS^−^) analyses were collected in acid-prewashed high-density polyethylene bottles without acidification. Aliquots for stable isotope analyses of oxygen (δ^18^O), hydrogen (δD), and carbon in dissolved inorganic carbon (δ^13^C_DIC_) were transferred into 2 mL septum screw-lid glass vials. Samples for δ^13^C_DIC_ analysis were preserved with 30 µL of saturated HgCl_2_ to inhibit microbial activity. All porewater samples were stored at 4 °C in a refrigerator until analyzed further. The water from the dissociated gas hydrates was pretreated and stored by following the same procedures as that used for storing porewater. Additionally, sediments from the porewater sampling depths were freeze-dried and frozen for grain size measurement and DNA-based analyses, respectively.

### Gas analysis, Porewater chemistry, grain size, and DNA extraction

Headspace gas samples were heated to 65 °C for 30 min to extract the gases [27]. Aliquots of headspace and dissociated hydrate gases were injected into an Agilent Technologies 7890 A gas chromatograph (GC; Agilent Technologies, USA) equipped with both a flame ionization detector and thermal conductivity detector to analyze the hydrocarbon composition (C_1_–C_6_) and CO_2_ concentrations. The GC conditions for the analysis were set as follows: inlet temperature, 230 °C; detector temperature, 250 °C; and oven temperature, increased from 35 °C (held for 5 min) to 195 °C at 20 °C/min. The stable carbon isotope in CH_4_ (δ^13^C_CH4_) and CO_2_ (δ^13^C_CO2_) and the hydrogen isotope in CH_4_ (δD_CH4_) were measured using a gas chromatograph/isotope ratio mass spectrometer (Isotech Laboratories Inc., USA). Carbon isotope ratios were calibrated against Vienna-Pee Dee Belemnite (V-PDB), and hydrogen isotopes were referenced to Vienna-Standard Mean Ocean Water (V-SMOW) with an analytical reproducibility of ± 0.1‰ for carbon isotopes and ± 2‰ for hydrogen isotopes.

Onboard porewater was analyzed for salinity, Cl^−^, and alkalinity. Salinity was determined using a portable reflectometer (Fisher Scientific, USA), and Cl⁻ concentration was determined using the Mohr titration method with 0.1 M AgNO_3_ by following the method described in the ODP Technical Note 15 [28]. Alkalinity was measured through titration with 0.02 M HCl. Ammonium (NH_4_^+^), phosphate (PO_4_^3−^), and HS^−^ concentrations were determined using a Shimadzu UV-2450 spectrophotometer (Shimadzu Corporation, Japan) at the Korea Institute of Geoscience and Mineral Resources (KIGAM). SO_4_^2−^ concentration was analyzed using a Dionex ICS-1500 ion chromatograph (Thermo Scientific, USA) at the KIGAM. Salinity calibration was performed using IAPSO Seawater (34.99 PSU) to ensure reproducibility (< 2% for alkalinity and < 0.5% for Cl^−^). The stable isotopes of hydrogen (δD) and oxygen (δ^18^O) were quantified at Beta Analytic using a Thermo Fisher Delta V mass spectrometer (Thermo Fisher, Germany). Carbon isotopes (δ^13^C) were analyzed at Oregon State University using a Finnigan DELTA-Plus mass spectrometer (Thermo Finnigan, Germany) with a reproducibility of ± 0.5‰ for δD, ± 0.1‰ for δ^18^O, and < ± 0.03‰ for δ^13^C.

Grain-size analysis was performed by washing the freeze-dried sediments, followed by desalination and treatment with 10% H_2_O_2_ to remove organic matter before sieving through a 63-µm mesh. Particle sizes were measured using a Sedigraph 5000D laser granulometer (Micromeritics, USA) and classified as sand (> 63 μm), silt (4.0–63 μm), or clay (< 4.0 μm). DNA was extracted from 0.25 g of sediment using the DNeasy PowerSoil Pro Kit (QIAGEN, Germany) by following the manufacturer’s protocol. DNA concentrations were quantified using the Qubit dsDNA High-Sensitivity Assay (Invitrogen, USA).

### Metabarcoding library and bioinformatics

The extracted DNA was prepared for metabarcoding-based sequencing using a two-step PCR method, which comprised an initial amplicon PCR followed by an index PCR. The primer sequences used and specific conditions for the two-step PCR have been previously described [10]. Briefly, the first amplicon PCR was performed in triplicate reactions using a KAPA HiFi HotStart ReadyMix PCR Kit (KAPA BioSystems, USA). The sequencing primers targeted the prokaryotic 16 S rRNA gene (V3–V4 region; 341 F: 5´-TCGTCGGCAGCGTCAGATGTGTATAAGAGACAG [Illumina adapter]– CCTACGGGNGGCWGCAG-3´; 806R: 5´-GTCTCGTGGGCTCGGAGATGTGTATAAGAGACAG [Illumina adapter]–GACTACHVGGGTATCTAATCC-3´), following Illumina’s adapter design. The obtained PCR amplicons were purified using a QIAquick PCR Purification Kit (Qiagen, USA). The second index PCR was performed using these amplicons according to the instructions provided by Illumina. During library preparation, no-template controls were included in both amplicon and index PCR steps and confirmed to yield no visible PCR product. Although these controls were not sequenced, potential contaminants were further minimized by applying sequence-based quality filtering and chimera removal.

Sequencing data analysis was performed using the Mothur software package (v. 1.40.5) [29] by following the MiSeq standard operating procedure (SOP) [30]. In total, 725,033 sequences were obtained for the 16 S rRNA gene after performing sequence quality filtration, which involved correcting amplification and sequencing errors, removing singletons, and performing random subsampling of sequences using the commands specified in the Mothur SOP. Prior to random subsampling, taxonomic classification was assigned using the “classify.seqs” command; further, non-prokaryotic sequences such as chloroplasts, mitochondria, eukaryotes, and unknown lineages were removed using the “remove.lineage” command. On average, each sample yielded 36,252 sequences (± 2,921), and the sequence count for each sample was normalized to 10,000 sequences through random subsampling.

The filtered sequences were clustered into amplicon sequence variants (ASVs) to analyze microbial alpha and beta diversity and community composition. Alpha diversity was assessed using abundance-unweighted species richness indices (Chao1 and ACE), which were calculated based on the ASVs using the “rarefaction.single” command. Beta diversity was assessed by generating a phylip-formatted distance matrix using Bray–Curtis dissimilarity. Non-metric multidimensional scaling (NMDS) was applied to visualize the differences in beta diversity using the “nmds” command. Statistical separation of beta diversity was evaluated using analysis of molecular variance (AMOVA) using the “amova” command. Linear discriminant analysis (LDA) with linear discriminant analysis effect size (LEfSe) [31] was performed using the “lefse” command to identify biomarkers by calculating LDA scores based on the relative abundance of statistically significant ASVs between cores.

Further statistical analyses were performed in R (v.4.2.0) using the “stats” package for correlation analysis and principal component analysis (PCA), whereas ASV distribution was visualized through heatmaps generated using the “ComplexHeatmap” package. Permutational multivariate analysis of variance was performed using the Euclidean distance method, which was implemented using the “vegan” package. Additionally, the rare ASVs were filtered, and bacterial functional potential was predicted using “PICRUSt2” [32] and classified based on the Kyoto Encyclopedia of Genes and Genomes database. Phylogenetic analysis was performed using the Molecular Evolutionary Genetics Analysis (MEGA, version 11) software [33]. Representative ASVs and reference sequences that were obtained from the National Center for Biotechnology Information GenBank were selected for analysis and aligned using the ClustalW algorithm implemented in MEGA. The alignment was manually inspected for removing ambiguities and trimming gaps to ensure accuracy. A phylogenetic tree was constructed using the Neighbor-Joining algorithm and the default MEGA parameters. The statistical support for the tree topology was evaluated through bootstrapping with 1,000 replicates. The final phylogenetic tree was visualized and annotated in MEGA. Branch lengths and bootstrap values were incorporated to interpret the evolutionary relationships among the selected ASVs and reference sequences.

## Results

### Geochemical properties

Gas and porewater samples extracted from sediment cores (Site P01, P03, and P04) displayed largely uniform geochemical characteristics, except for the deeper section of Site P03 below 2.5 mbsf (meter below the seafloor) (Fig. S1). This environmental heterogeneity was further illustrated by the PCA pattern, which accounted for 66.28% of the total variance, with PC1 and PC2 contributing 38.10% and 28.18%, respectively. Although no dominant variables were identified for these axes, most samples aligned along depth gradients, with variables such as SO_4_^2−^, alkalinity, PO_4_^3−^, HS^−^ closely associated with depth changes. PCA patterns appeared consistent with geochemical variables in Site P01 and P04, while Site P03 demonstrated pronounced heterogeneity, especially in its vertical depth profile (Fig. S1). Concentrations of salinity (33 ~ 34 PSU) and Cl^−^ (541 ~ 551 mM) at Sites P01 and P04 exhibited relatively constant values throughout the core. In contrast Site P03 showed a substantial decline in these values (salinity from 33 to 26 PSU and Cl^−^ from 551 to 450 mM, below 2.3 mbsf, corresponding to depths where gas hydrates were visually identified during the expedition (Fig. 1B). Dissociated gas hydrates in Site P03 presented significantly lower salinity (2.4 ~ 6.3 PSU) and Cl^−^ concentrations (40 ~ 100 mM) compared to surrounding porewater (Table S2). These observations indicate that hydrate dissociation releases water with low salinity and Cl^−^ concentration into porewater, which results in anomalous geochemical signatures [34, 35]. Additionally, this evidence supports the identification of hydrate-bearing intervals at Site P03.

**Fig. 2:**
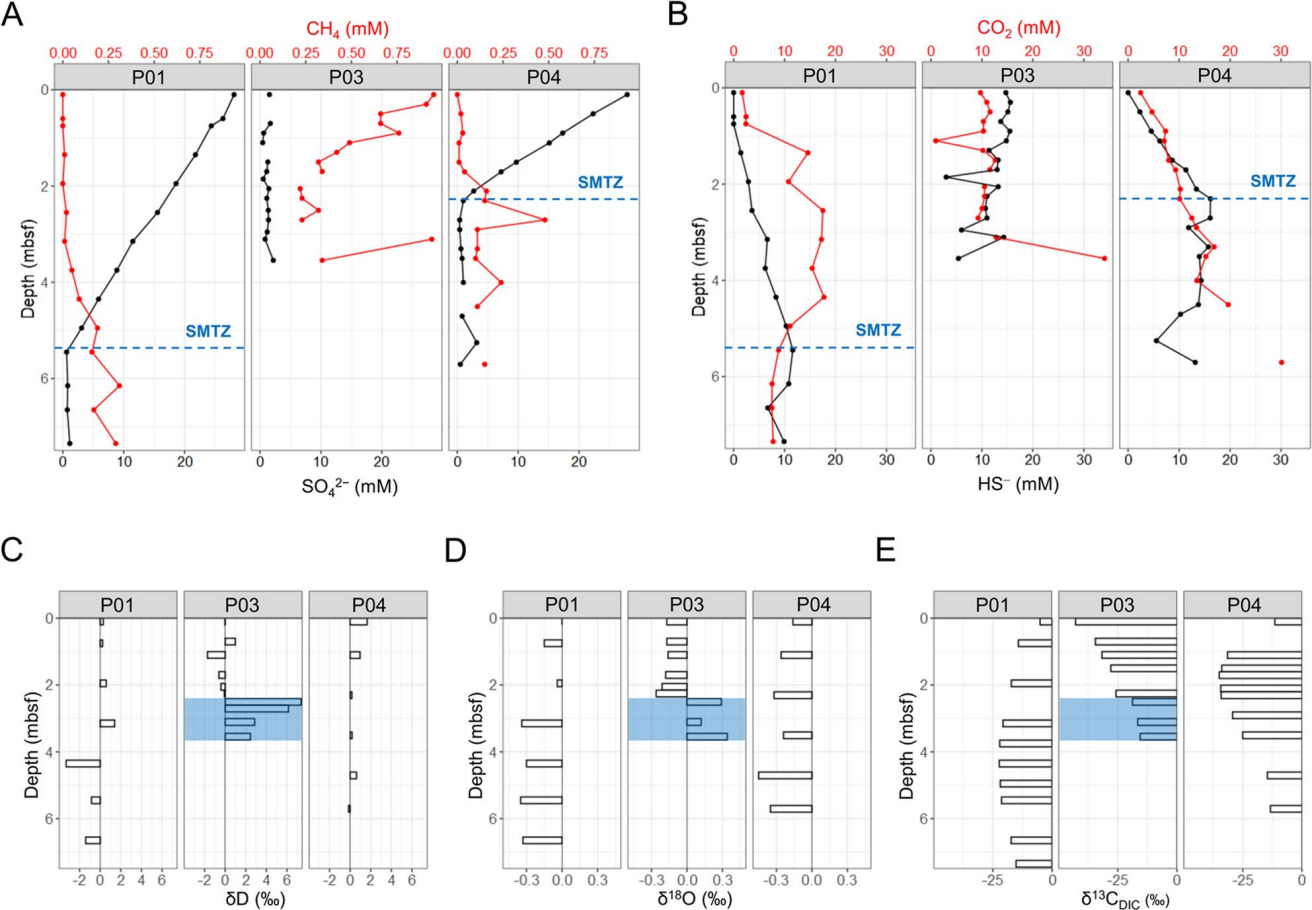
Depth profiles of (**A**) SO_4_^2−^ and CH_4_ and (**B**) HS^−^ and CO_2_ in porewaters, and stable isotope compositions of (**C**) hydrogen (δD), (**D**) oxygen (δ^18^O), and (**E**) dissolved inorganic carbon (δ^13^C_DIC_). Depth is shown in meters below seafloor (mbsf). The sulfate–methane transition zone (SMTZ) is indicated by dashed lines in panels (**A**) and (**B**) for Sites P01 and P04. The SMTZ at P03 is inferred to occur near the sediment surface (< 0.5 mbsf) based on geochemical trends and hydrate occurrence, but it was not directly determined due to the lack of porewater data in that interval. SO_4_^2−^ and HS^−^ are shown in black, whereas CH_4_ and CO_2_ are shown in red. The shaded area in Site P03 (panels **C**–**E**) denotes the hydrate-bearing interval with measured values listed in Table S2. In panels (**A**) and (**B**), unlinked points indicate depth intervals where data were not measured

The variations in SO_4_^2−^ and CH_4_ concentrations further highlighted the differences in SMTZ depths across the sites (Fig. 2A). In this study, we defined the SMTZ as the depth interval where SO_4_^2−^ concentrations approached depletion concomitant with a sharp increase in CH_4_ concentrations. At Site P01 (non-chimney structure), the SMTZ was located at approximately 5.3 mbsf. In contrast, the SMTZ was interpreted as near-surface (< 0.5 mbsf) at Site P03 and as 2.3 mbsf at Site P04, respectively. As described above, gas hydrates were recovered from Site P03. Hydrate dissociation released freshwater, as evidenced by the markedly low salinity and Cl^−^ concentrations of dissociated hydrate water. This process also enhanced CH_4_ flux, and the upper sediments at P03 consequently showed elevated CH_4_ and depleted SO_4_^2−^, supporting the interpretation of a shallow SMTZ at this site. These differences indicate that geological structures influence the vertical migration of CH_4_ and, consequently, the position of the SMTZ. In the SMTZ, SO_4_^2−^ reduction is microbially coupled with CH_4_ oxidation, leading to the production of HS^−^ and CO_2_ (Fig. 2B). The resulting CO_2_ is largely derived from anaerobic CH_4_ oxidation. Due to its higher solubility compared to CH_4_, CO_2_ tends to accumulate more in porewater, particularly in areas with intensive CH_4_ flux. These variations highlight the interplay between geological structures, CH_4_ flux, and CO_2_ production in determining SMTZ positioning.

**Fig. 3:**
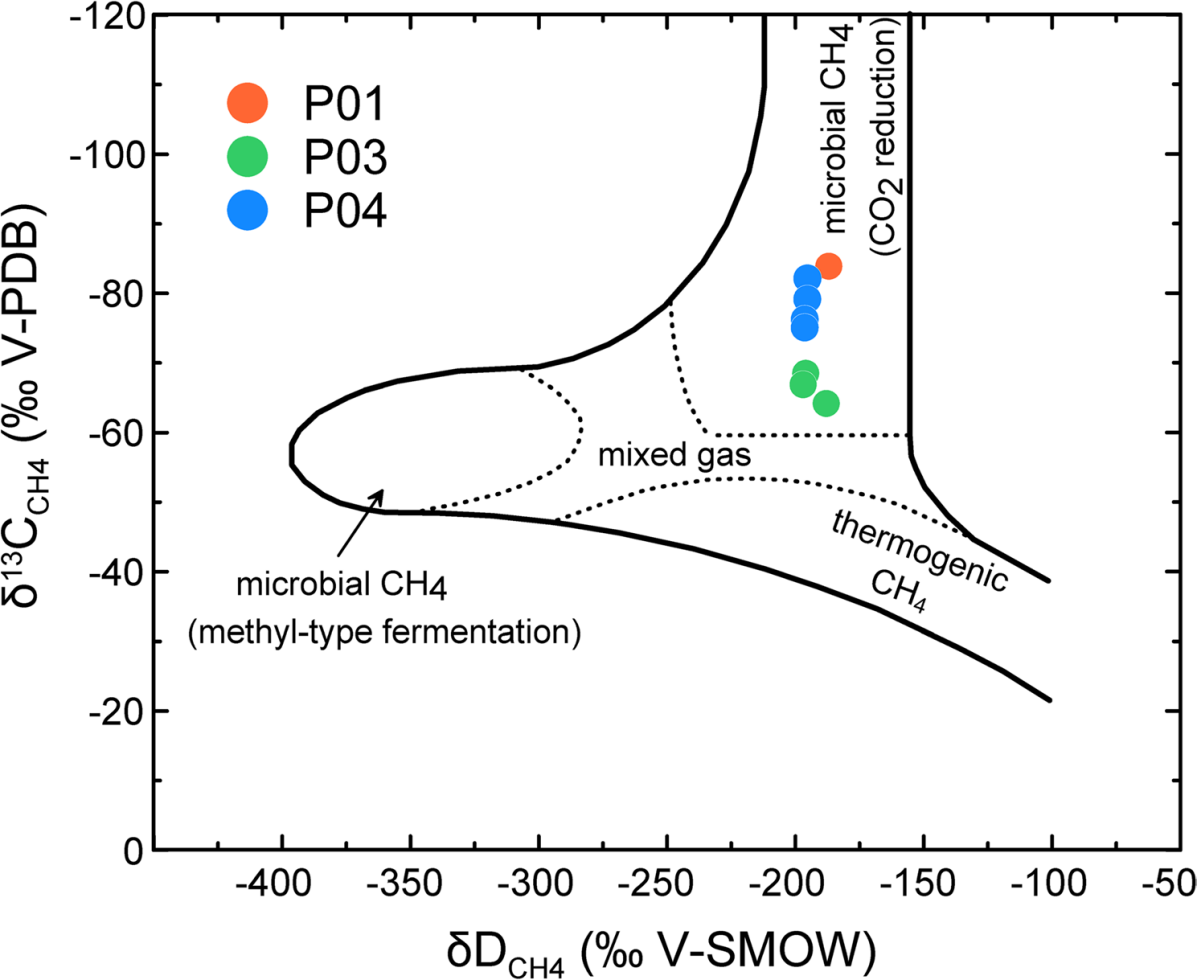
Cross plot of δ^13^C_CH4_ (‰, relative to VPDB) versus δD_CH4_ (‰, relative to VSMOW) for headspace gas samples. Dashed areas indicate fields for thermogenic CH_4_, microbial CH_4_ (via CO_2_ reduction or methyl-type fermentation), and mixed gas origin [36]. Symbols represent sites (P01, P03, P04). VPDB = Vienna Pee Dee Belemnite; VSMOW = Vienna Standard Mean Ocean Water

Isotopic analyses of porewater samples (δD, δ^18^O, and δ^13^C_DIC_) revealed site-specific patterns. Site P03 showed distinct δD and δ^18^O enrichments below 2.3 mbsf, consistent with hydrate dissociation, whereas P01 and P04 remained relatively stable (Fig. 2CD). This indicates that chimney cores exhibited more pronounced isotopic anomalies than the non-chimney core. All sites exhibited minimum δ^13^C_DIC_ near the SMTZ, but the depths of minima differed, occurring deeper in P01 and shallower in P03 and P04 (Fig. 2E). Headspace gas isotopes (δ^13^C_CH4_, δD_CH4_, and δ^13^C_CO2_) showed site-specific minima near the SMTZ (Table S3), supporting active AOM [1, 2]. Chimney cores (P03, P04) displayed shallower isotope minima than the non-chimney core (P01), indicating enhanced near-surface CH_4_ consumption. All values fell within the range of microbial CH_4_ origin via CO_2_ reduction [36] (Fig. 3), consistent with previous surveys in the Ulleung Basin [13, 14, 22, 23].

Despite these geochemical differences, the cores exhibited significantly similar grain-size patterns (*P* >0.05; Fig. S2). All sediment cores predominantly consisted of clay-rich silt, which is a common sediment-type in various marine environments including deep-sea basins. The vertical distribution of these sediments remained consistent from the top to the bottom of the cores (Fig. S2). This indicates that the environmental conditions, sediment transport dynamics, and resource potential remained stable throughout the sediment deposition. Generally, eDNA concentration is higher in the upper layers of the sediment cores compared with those of the lower layers. This trend was particularly evident at Site P01 (Fig. S3). Relatively higher eDNA concentrations in the upper sediment layer may reflect enhanced microbial signal associated with fresh organic matter input [6], although this does not directly quantify biological activity. In contrast, Sites P03 and P04 exhibited relatively consistent eDNA concentrations at all areas of the core. This result indicates stable and conserved biological activity from top to bottom.

### Microbial diversity and community composition

**Fig. 4:**
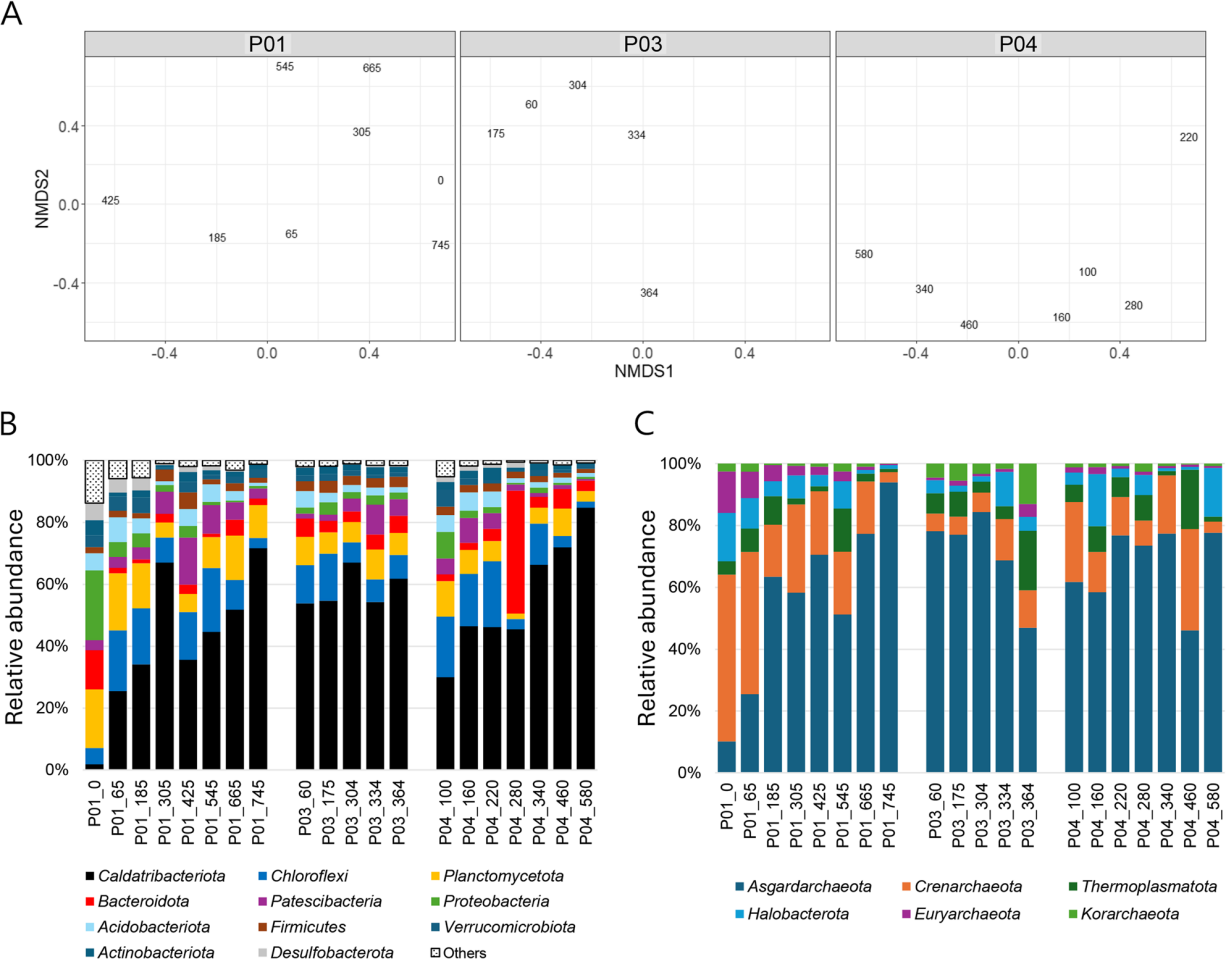
Microbial diversity and community composition. (**A**) Non-metric multidimensional scaling (NMDS) ordination of microbial communities. (**B**) Relative abundance of bacterial phyla and (**C**) archaeal phyla across depth intervals. Labels on NMDS plots denote the sampled depths (cmbsf) for each core. Microbial taxa with a relative abundance of less than 1% within each sample were classified as other groups

In the alpha diversity analysis, indices of species richness (Chao and Ace) showed strong and significant negative correlations with depth at P01 (Chao: −0.89, *P* < 0.05; Ace: −0.89, *P* < 0.05) and P04 (Chao: −0.82, *P* < 0.05; Ace: −0.77, *P* < 0.05) (Fig. S4). This indicates that the upper sediment layers at Sites P01 and P04 are more enriched with microbial species than the deeper sediment layers. In contrast, correlations at Site P03 were weak and not significant (Chao: −0.41, *P* > 0.05; Ace: −0.41, *P* > 0.05), suggesting no clear depth-related trend in microbial richness at this site. Furthermore, NMDS ordination of beta diversity showed that microbial communities were not significantly differentiated across most sediment samples, except for the pairwise comparison between Sites P03 and P04 (Fig. S5). In particular, the upper layer of P03 sediments (0.60–3.34 mbsf) exhibited significant differentiation from the other sediment layers (AMOVA, *P* < 0.05), consistent with the NMDS pattern (Fig. 4A). This indicates unique community characteristics within this interval.

Further analysis of community composition identified the specific taxa present in the microbial communities and their relative abundances, which provide deeper insights into the microbial diversity. The bacterial communities primarily comprised the phyla *Caldatribacteriota* (51.3%), *Chloroflexi* (11.1%), *Planctomycetota* (9.0%), and *Bacteroidota* (5.9%). Moreover, their distribution exhibited distinct spatial patterns at various sediment depths (Fig. 4B). For example, *Caldatribacteriota* was the dominant bacterial group, and its abundance declined sharply from the lower to upper layers at Sites P01 and P04, whereas its abundance remained relatively consistent throughout Site P03. Notably, all identified *Caldatribacteriota* sequences belonged to the JS1 clade at the class level (Fig. S6), which corresponds to an uncultured JS1-related lineage at the family level that was consistently abundant at Site P03 (Table S4). Other major lineages at the family level, such as *Anaerolineaceae* (within *Chloroflexi*) and *Planctomycetaceae* (within *Planctomycetota*), also displayed pronounced depth-related variations at Sites P01 and P04 but remained more uniform at Site P03 (Table S4). Compared with these bacterial patterns, archaeal communities exhibited a simpler composition (Fig. 4C). The predominant archaea identified were *Asgardarchaeota* (66.1%) and *Crenarchaeota* (17.4%) (Fig. 4C). Furthermore, the *Asgardarchaeota* sequences were further classified into two major subclasses: *Odinarchaeia* (37.2%) and *Lokiarchaeia* (28.8%). At the family level, these correspond to *Odinarchaeaceae* and *Lokiarchaeaceae*, respectively, while the *Crenarchaeota* were primarily represented by *Thermoproteaceae* (Table S4). In particular, *Lokiarchaeaceae* and *Methanofastidiosales*-related lineages at the family level showed site-specific variation at Sites P01 and P04 but were more stable across depths at Site P03 (Table S4). Particularly, JS1 and *Lokiarchaeia* (previously known as Marine Benthic Group B) are the dominant microbial populations in various methane-rich subseafloor environments [37–41]; this concurs with our findings in the Ulleung Basin [20].

### SMTZ profiles with predicted microbial pathways and population dynamics

**Fig. 5:**
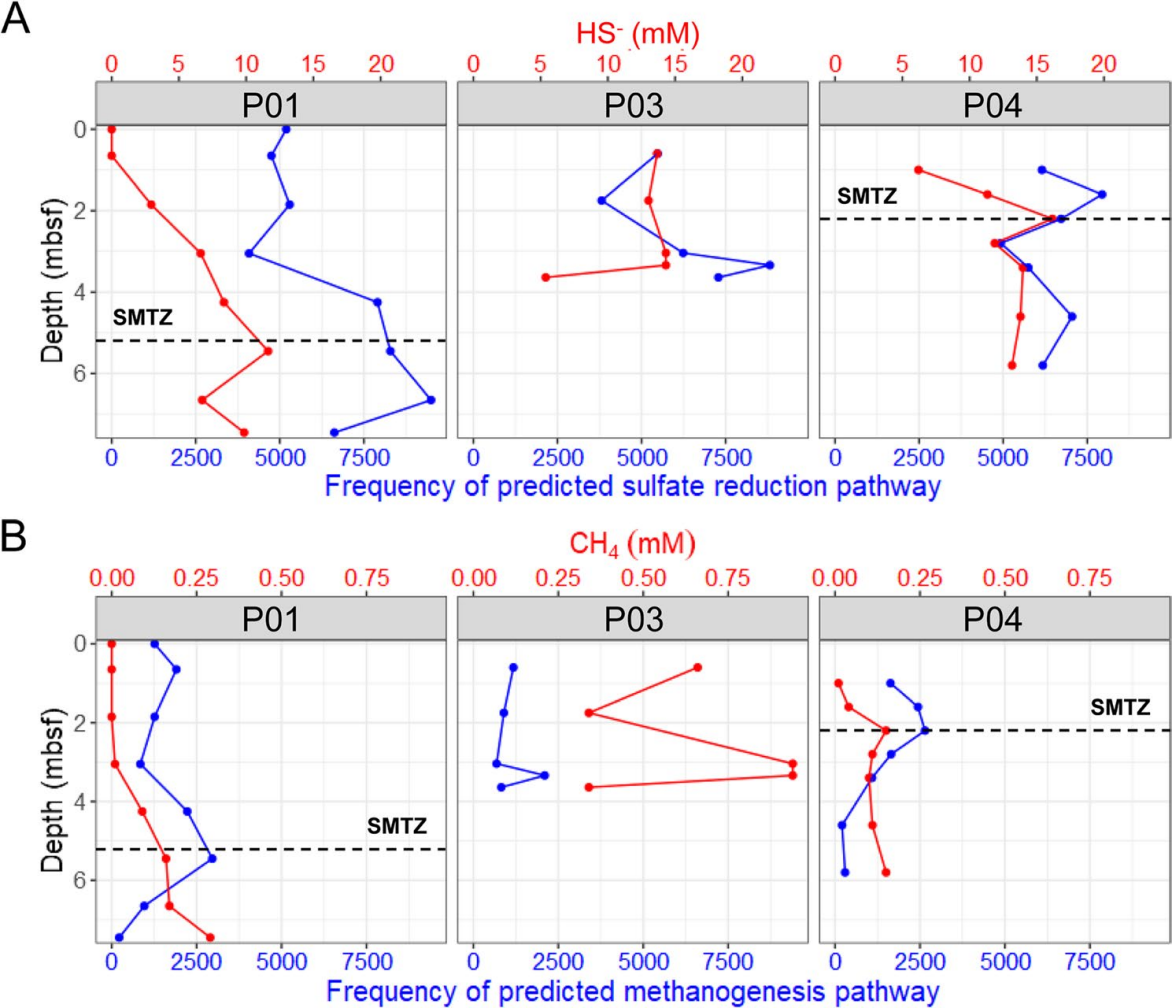
Depth profiles of (**A**) hydrogen sulfide (HS^−^) concentrations compared with predicted sulfate reduction pathways and (**B**) methane (CH_4_) concentrations compared with predicted methanogenesis pathways. The sulfate–methane transition zone (SMTZ) is indicated by dashed lines. Predicted pathways were inferred from PICRUSt2 and are expressed as relative gene frequencies (normalized counts). Pathways were classified using KEGG database annotations

The functional genes associated with microbial SO_4_^2−^ reduction and methanogenesis were predicted using PICRUSt2 and the Kyoto Encyclopedia of Genes and Genomes database. The frequency of the predicted SO_4_^2−^ reduction pathway was significantly higher than that of the methanogenesis pathway at all sites (Fig. 5). Notably, the predicted frequency of SO_4_^2−^ reduction closely resembled the HS^−^ profile (Fig. 5A). Similarly, the predicted frequency of the methanogenesis pathway showed a distribution pattern that was similar to that of CH_4_ concentration above the SMTZ. However, methanogenesis declined sharply below the SMTZ. This trend was particularly evident at Sites P01 and P04 (Fig. 5B).

SO_4_^2−^ concentration decreases within the SMTZ, whereas HS^−^ concentration increases due to the activity of SRB. Among the microbial communities, various populations exhibited positive correlation with SO_4_^2−^ concentration (ρ >0.5). However, only an unclassified subclass of *Halobacterota*, *Planctomycetes*, *Methanobacteria*, and *Desulfobacteria* simultaneously displayed negative correlation with HS^−^ concentration (ρ < −0.5) (Fig. S7). This result highlights the potential metabolic capabilities of these communities and their ecological roles in response to SRB. Among these, *Desulfobacteria* are the quintessential SRB as they are highly specialized in SO_4_^2−^ reduction in anaerobic and SO_4_^2−^-rich environments [42]. Moreover, the SMTZ acts as a critical zone for highly concentrated CH_4_ consumption and CO_2_ production. Among the microbial populations that were associated with CO_2_ and CH_4_ concentrations (Fig. S7), the relative abundance of *Odinarchaeia* positively correlated with CH_4_ (ρ >0.5), whereas *Desulfobacteria* and the unclassified *Halobacterota* subclass showed negative correlation (ρ < −0.5). Furthermore, *Clostridia* and JS1 showed positive correlation with CO_2_ concentration (ρ >0.5), whereas *Planctomycetes* and *Phycisphaerae* showed negative correlation (ρ < −0.5).

**Fig. 6:**
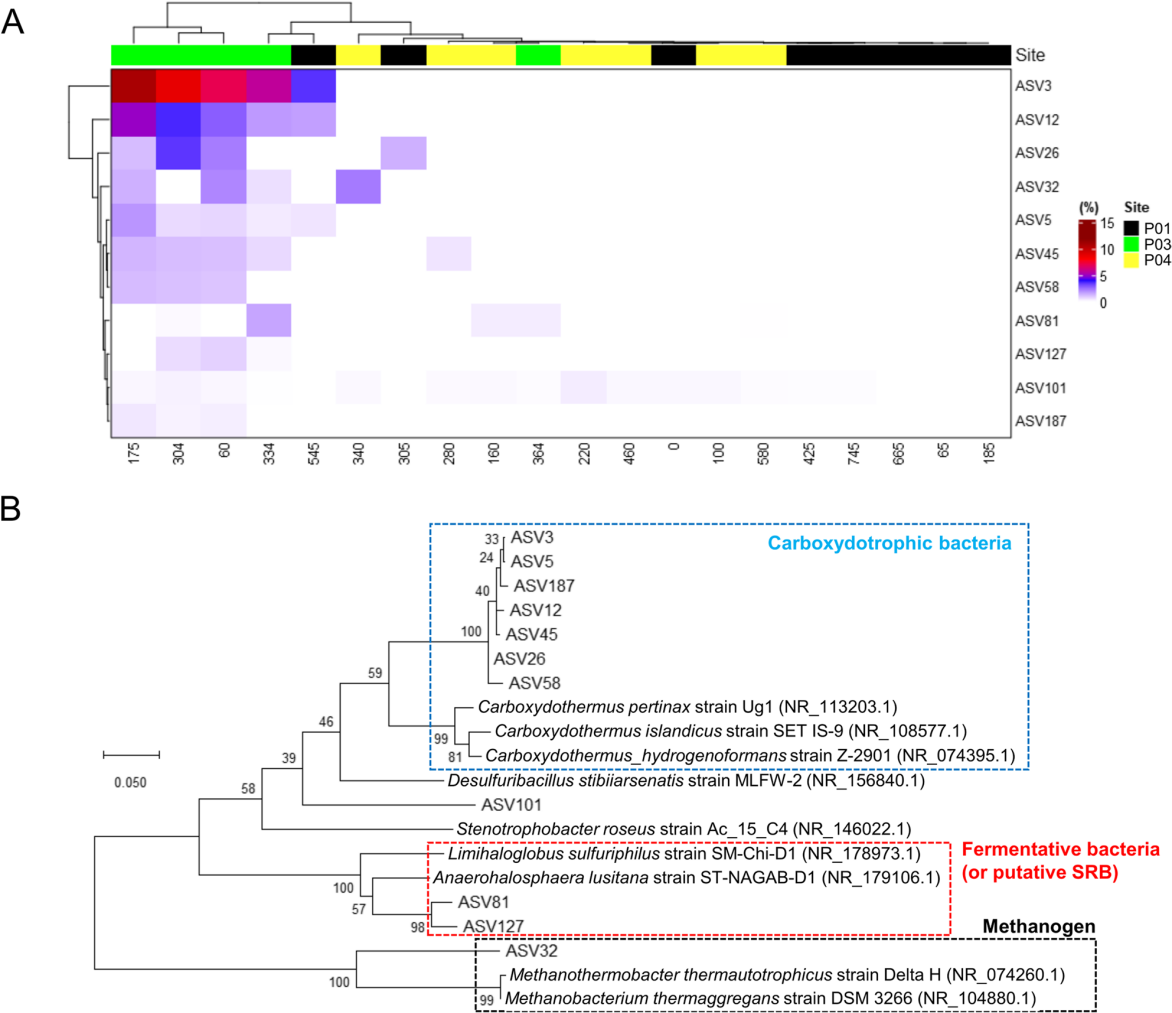
Microbial indicator analysis using amplicon sequence variants (ASVs) selected based on linear discriminant analysis (LDA) scores. **A** Distribution of microbial ASV indicators across sites (P01, P03, P04) visualized using a heatmap. The numbers shown on the horizontal axis correspond to sediment depth (cmbsf). **B** Phylogenetic tree of the selected ASVs along with reference sequences, highlighting microbial groups, including methanogens, fermentative bacteria (putative SRB), and carboxydotrophic bacteria. The tree was constructed using the maximum likelihood method, and bootstrap values (1,000 replicates) are shown at branch nodes

Microbial indicators were determined using LEfSe, and LDA scores were calculated based on the relative abundance of ASVs across sediment cores (Sites P01, P03, and P04). Among the 55,630 microbial ASVs that were analyzed, 11 exceeded the significance threshold (LDA score > 3, *P* < 0.05) after pairwise comparisons between the three cores. These ASVs showed significant association with a single core in both comparisons. Among these 11 ASVs, one was common to all sites, whereas the remaining 10 were specific to Site P03 (Table S5). The relative abundance of these microbial ASVs was visualized using a heatmap, and their taxonomy at the genus level was classified using the SILVA database. The heatmap of Site P03-specific ASVs highlighted their prominence in the upper sediment layers (0.60–3.34 mbsf) (Fig. 6A). This result aligned with the clustering pattern observed in the beta diversity (NMDS) analysis (Fig. 4A). Among the 10 specific ASVs at Site P03, seven were identified as carboxydotrophic bacteria, two as fermentative bacteria, and one as a methanogen (Fig. 6B).

## Discussion

### Geochemical signatures of gas hydrate dissociation

Our results reveal how gas hydrate-related CH_4_ migration drives localized geochemical alterations and microbial responses, particularly within chimney structures. The chimney structures acted as critical conduits for CH_4_ migration to the surface and hotspots for geochemical processes. A comparison of the non-chimney and chimney sites suggested that higher CH_4_ fluxes in the chimney sites may be associated with shallower SMTZs. Additionally, SO_4_^2−^ concentrations decreased to 0 mM, and CH_4_ concentrations increased sharply near the SMTZ at Sites P03 and P04. Similarly, alkalinity increased near the SMTZ because of bicarbonate production during AOM but decreased below 2.3 mbsf at Site P03. This pattern, together with the significant decrease in salinity and Cl⁻ concentration, indicates the release of freshwater from gas hydrates during dissociation. Isotopic analyses corroborated these observations. These geochemical conditions explain why the SMTZ at Site P03 was interpreted near the surface. Hydrate dissociation generated freshwater anomalies in the deeper hydrate-bearing interval and simultaneously enhanced CH_4_ flux, causing elevated CH_4_ and SO_4_^2−^ depletion in the upper sediments, consistent with the formation of a shallow SMTZ. Along with elevated CO_2_ and HS⁻ levels resembling those below the SMTZ in P01 and P04, these results support the occurrence of shallow AOM at P03.

Sharp isotopic transitions at Site P03 indicate the presence of hydrate-bearing intervals. For example, both δD and δ^18^O values increase significantly below 2.3 mbsf, the depth at which gas hydrates were visually observed (Fig. 1B). This isotopic enrichment could result from the release of isotopically heavier water (enriched in deuterium and δ^18^O) that was preferentially incorporated during hydrate formation into the surrounding environment upon its dissociation [34, 35]. Such isotopic signals from hydrate dissociation may occur either during core recovery or as an ongoing in situ process within the sediments. Although hydrate dissociation can also occur during core recovery due to temperature and pressure changes, several lines of evidence indicate that the signals at Site P03 primarily reflect in situ processes. Below ~2.3 mbsf, the combined porewater anomalies (low salinity and Cl⁻ together with enriched δD and δ^18^O) are best explained by long-term in situ hydrate dissociation rather than recovery artifacts. Above ~2.3 mbsf, CH_4_ concentrations remained high while SO_4_^2−^ was relatively depleted (Fig. 2), reflecting strong upward CH_4_ flux and fluid advection through the chimney structure. However, HS⁻ and CO_2_ concentrations, together with CH_4_, were comparable to those observed below the SMTZ in other cores, suggesting that the redox regime remained similar to deeper sediments. This dual signal indicates that the anomalies at Site P03 reflect both sustained hydrate dissociation and CH_4_-driven fluid advection through the chimney structure.

The consistent grain-size data imply that geochemical variations among the cores do not appear to be influenced by sedimentological changes (Fig. S2). Instead, these variations are strongly associated with microbial activity. This highlights the essential role of microbes in regulating geochemical processes in hydrate-bearing sediments. Geochemical conditions in marine sediments significantly shape microbial community structure and diversity [10, 43]. Moreover, microbial distribution, diversity, and geochemical properties in methane hydrate-bearing environments are distinctly related to each other [1, 25, 44–47]. This underscores the interplay between geochemical dynamics and microbial activity in shaping sedimentary processes in hydrate-bearing systems. The results of our analysis were consistent with these findings and showed that the minimum δ^13^C_DIC_ values observed near the SMTZ are indicative of AOM. The consumption of CH_4_ by the microbes results in the incorporation of isotopically lighter carbon into the DIC pool. Consequently, the lowest δ^13^C_DIC_ value observed near the SMTZ at Site P03 reflects the highest intensity of AOM activity among the three sites.

### Microbial community dynamics and functional pathways in the SMTZ

Globally, SMTZ microbial communities are typically dominated by ANME archaea and their sulfate-reducing partners, *Deltaproteobacteria*, which together consume up to 90% of CH_4_ produced in deep sediments and thus play a vital role in carbon cycling [44, 48–50]. However, the dominance and activity of these groups vary depending on local environmental conditions [51]. For instance, in certain SMTZs such as those found in the Ulleung Basin, other microbial groups such as JS1 and *Lokiarchaeia* contribute to CH_4_ cycling, particularly in those regions where the ANME populations are sparse or absent [37–41]. These findings suggest that alternative microbial pathways may be linked to CH_4_ metabolism in deep subseafloor sediments [37, 39, 41]. Particularly, JS1 and *Lokiarchaeia* were consistently present at Site P03, highlighting the influence of gas hydrates on microbial community composition and distribution. JS1 is commonly associated with gas hydrates [45, 52], and it likely contributes to CH_4_ cycling through syntrophic interactions with the SRB. For example, JS1 may produce hydrogen via fermentation, which may be utilized by the methanogens or SRB [53]. Similarly, *Lokiarchaeia* is another dominant archaeal taxon that exhibits metabolic versatility, engaging in syntrophic interactions with methanogens or SRB [54]. This emphasizes its role in biogeochemical processes in hydrate-bearing sediments.

In the SMTZ, SO_4_^2−^ is consumed via SRB or AOM activity, which results in HS^−^ production. The relationship between SO_4_^2−^ depletion and HS^−^ accumulation defines the sulfide production zone, and it reflects the balance of dissolved sulfide species (H_2_S and HS^−^) under typical porewater pH conditions. SO_4_^2−^ reduction pathway was identified as the predominant pathway at all sites. The SRB such as *Desulfobacteria* showed strong correlations with SO_4_^2−^ and HS^−^ concentrations. The predictions of microbial functional potential based on PICRUSt2 analyses aligned with these patterns, which indicate that SO_4_^2−^ reduction pathway closely mirrors HS^−^ distribution in the SMTZ. Additionally, the predicted functional pathways have provided insights into key microbial processes that regulate CH_4_ cycling and carbon turnover in hydrate-bearing sediments. While PICRUSt2 provides predictive insights into microbial functional potential based on phylogenetic inference, it does not directly confirm gene expression or enzymatic activity. Thus, these predictions should be regarded as putative and provide only a preliminary overview of community functions. Future metagenomic or metatranscriptomic analyses will be required to validate the metabolic traits inferred here. Nevertheless, in our study, the predicted pathways, particularly those related to sulfate reduction and methanogenesis, demonstrated notable alignment with observed biogeochemical trends, including HS^−^ and CH_4_ concentrations. This congruence suggests that, when interpreted alongside comprehensive geochemical data, PICRUSt2 predictions can offer ecologically meaningful insights even in complex and underexplored environments such as gas hydrate-bearing sediments.

The isotopic composition of methane (δ^13^C_CH4_ and δD_CH4_) observed in this study indicates the occurrence of methanogenesis via CO_2_ reduction, thereby excluding a thermogenic source, which typically exhibits heavier δ^13^C_CH4_ (–50 to − 20‰) and δD_CH4_ (–275 to − 100‰) values [36]. Typically, the methanogens in the upper SMTZ utilize hydrogen or acetate derived from organic matter degradation in shallow sediments or nearby fermentation zones [55, 56]. Below the SMTZ, sulfate depletion is expected to foster conditions that are favorable for methanogenesis [55]. However, in this study, methanogenesis peaked near the SMTZ and decreased sharply below it at Sites P01 and P04, which may be attributed to factors such as substrate limitation, unfavorable growth conditions, or competition with other microbial groups. Notably, the known microbial competitors such as *Deltaproteobacteria* and *Gammaproteobacteria* that utilize alternative electron acceptors such as iron or manganese [57] were not detected at significant levels below the SMTZ. This suggests that CH_4_ migration and accumulation rather than in situ biogenic production play a more prominent role in this zone of the Ulleung Basin. In contrast, the results from Site P03 suggest that CH_4_ consumption and intensified SO_4_^2−^ reduction are driven by increased CH_4_ flow from dissociated gas hydrates.

### Chimney-specific microbial indicators

The unique microbial indicators identified at Site P03 were determined through the LEfSe analysis based on significantly enriched ASVs. These indicators comprised carboxydotrophic bacteria, fermentative bacteria, and one methanogen. These microbial groups reflect the distinct geochemical conditions created by gas hydrate formation, which include elevated CH_4_ flux and changes in carbon substrate availability. Carboxydotrophic bacteria likely facilitate carbon monoxide oxidation, which contributes to organic matter turnover [58], whereas fermentative bacteria aid in breaking down complex organic compounds into simpler metabolites. Although a methanogen was detected as an indicator, it was not overwhelmingly represented compared to the carboxydotrophic bacteria ASVs, and the predicted functional pathways did not suggest methanogenesis as a dominant process at Site P03. Instead, isotopic and geochemical profiles demonstrated that AOM was the prevailing pathway in the SMTZ, particularly at Site P03, where δ^13^C_DIC_ values indicated the strongest AOM signal among the three cores. The presence of methanogens therefore likely represents localized micro-niches supported by fermentative substrates, rather than widespread activity. The prominence of these specific microbial taxa at Site P03 highlights their specialization and ecological roles in driving CH_4_ cycling. Additionally, it shows that the chimney site is a key area for studying microbial processes in hydrate-bearing sediments. Together, these chimney-specific microbial indicators point to the localized impact of gas hydrate dynamics on SMTZ depth and microbial function, setting the stage for broader ecological interpretations.

## Conclusions

This study offers integrated insights into how geochemical gradients and gas hydrate proximity shape microbial composition and function in CH_4_-rich marine sediments. Firstly, this study has revealed the differences in microbial community composition across the geochemical zones and particularly between the chimney and non-chimney sites. We have highlighted the differences in microbial taxa and their metabolic roles in CH_4_ cycling and SO_4_^2−^ reduction. Notably, we have elucidated the unique microbial indicators that emphasize the distinct community adaptations in CH_4_-rich environments. Secondly, we have investigated the manner in which the microbial consortia mitigate CH_4_ emissions through the coupling of CH_4_ oxidation and SO_4_^2−^ reduction. The traditional ANME–SRB partnerships appear to be less dominant in the Ulleung Basin and alternative taxa such as JS1 and *Lokiarchaeia* may play significant roles in CH_4_ cycling. These findings underscore the potential for diverse microbial pathways to regulate CH_4_ dynamics in methane-rich subseafloor environments. Thirdly, we have explored the impact of gas hydrate proximity on microbial communities. The unique microbial taxa enriched in this hydrate-affected zone reflect the distinct geochemical conditions and highlight the ecological roles of these organisms in CH_4_ cycling and carbon turnover.

In conclusion, this study presents a comprehensive understanding of microbial community dynamics and their interactions with geochemical gradients in the Ulleung Basin by integrating the metabarcoding approach with biogeochemical analyses. Moreover, the predicted metabolic pathways highlight the integration of various microbial processes in the CH_4_-rich environment. These findings contribute to a broader understanding of CH_4_ dynamics in marine sediments, a key component of the global carbon cycle with potential (or indirect) implications for climate change and ocean health. This study demonstrates that gas chimneys play a pivotal role in subseafloor carbon sequestration and biogeochemical feedback mechanisms, as illustrated by hydrate dissociation at Site P03, where freshening signals and elevated methane flux led to a shallow SMTZ and intensified AOM activity. Our observations at Site P03 therefore provide a site-specific example of how hydrate instability can alter microbial and geochemical processes. While we did not attempt to quantify future warming effects, these findings suggest that chimney structures with hydrate dissociation represent sensitive environments that warrant further investigation under changing ocean conditions. Potential primer-specific biases in archaeal coverage with the universal V3–V4 set cannot be excluded. However, assessing primer performance was beyond the scope of this study, and our interpretations are supported by concordant geochemical and isotopic evidence for active AOM at the SMTZ. While our study provides valuable insights into microbial community function using predictive tools, integrating direct approaches will be essential to confirm gene expression and metabolic activity in gas hydrate-bearing environments.

## Supplementary Information

Supplementary Material 1

## Data Availability

The obtained metabarcoding sequences have been submitted to the Sequence Read Archive of National Center for Biotechnology Information under the accession number PRJNA1219790. The metadata associated with the metabarcoding data used in this study are provided in the Supplementary information.
