# Supplementary material for: Methane dynamics in gas chimneys linking geochemical and microbial methane cycling in the Ulleung basin
Source: BMC Microbiol. 2025 Nov 22;26:8. doi: 10.1186/s12866-025-04547-7 (PMC12777270; doi:10.1186/s12866-025-04547-7)
Supplement: Supplementary file 1 — Supplementary Material 1 [file 12866_2025_4547_MOESM1_ESM.docx]

1. Description of Ulleung Basin

The East Sea is a semi-enclosed marginal sea in the northwestern Pacific Ocean and is bordered by Korea, Japan, and Russia. It comprises three major basins, the Japan, Yamato, and Ulleung basins, each exceeding 2,000 m in depth [1]. Particularly, the Ulleung Basin, which is located in the southwestern part of the East Sea near the Korean Peninsula, is influenced by upwelling and eddies that enhance biological and geochemical processes; hence, it exhibits higher biological productivity compared with those of the Yamato and Japan basins [2]. Furthermore, these processes are closely linked to the surface sediments of the Ulleung Basin, which are enriched with organic carbon and manganese oxides. Under suboxic conditions, this sediment composition facilitates carbon oxidation pathways and nutrient cycling through microbial processes, which further enhances the geochemical dynamics in the Ulleung Basin [3]. Additionally, multi-channel seismic data have indicated the abundant presence of acoustic chimneys in this basin. These structures serve as gas migration pathways from deep sediment layers to the sea floor where they potentially feed massive gas hydrate accumulations [3-6]. These unique sedimentary and geophysical features provide ideal conditions for methane hydrate formation and stability in this basin [7]. Thus, the Ulleung Basin serves as a natural laboratory for studying the complex interplay of biological and geological processes in methane-rich marine sediments [8]. As a marginal basin in the temperate zone, the Ulleung Basin also experiences pronounced seasonal stratification, vertical mixing, and episodic changes in water column structure [9-11]. These hydrographic dynamics, along with its high organic carbon load and gas hydrate occurrence, suggest that the basin may be particularly sensitive to environmental changes such as ocean warming [10], hydrate destabilization, and altered methane flux [12].

2. Seismic profiles across the study sites

The multi-channel seismic (MCS) profiles were acquired in 2000 and 2005 in the Ulleung Basin of the East Sea during gas hydrate research conducted by the Korea Institute of Geoscience and Mineral Resources (KIGAM) using the R/V *Tamhae II*. The seismic source consisted of an array of six air guns with a total volume of 1035 in^3^ operated at 2000 psi. For data acquisition, the 2000 survey used an 84-channel streamer, whereas the 2005 survey employed a 240-channel streamer with a group interval of 12.5 m and a shot interval of 25 m.

In the seismic profiles (Graphic Abstract), the vertical axis represents two-way travel time (TWT, seconds), corresponding to the time required for the seismic wave to travel from the source to a reflector and back to the receiver. The horizontal axis is shown in common depth point (CDP) numbers, which represent the spatial sampling of seismic traces along the survey line. Although the nominal CDP spacing in the acquisition geometry was 6.25 m, the data were processed with a spacing of 12.5 m.

The reference profile across Site P01 shows parallel and continuous reflections with no evidence of chimney structures or fluid-escape features, representing background stratigraphy unaffected by fluid migration. In contrast, the profile across Site P03 displays a chimney structure in the central part of the section, characterized by disrupted and attenuated reflections (acoustic blanking), interpreted as evidence of upward migration of free gas and/or fluids disturbing the overlying strata. A similar feature is observed at Site P04, where disrupted and weakened reflections also indicate upward fluid/gas migration pathways comparable to those at P03


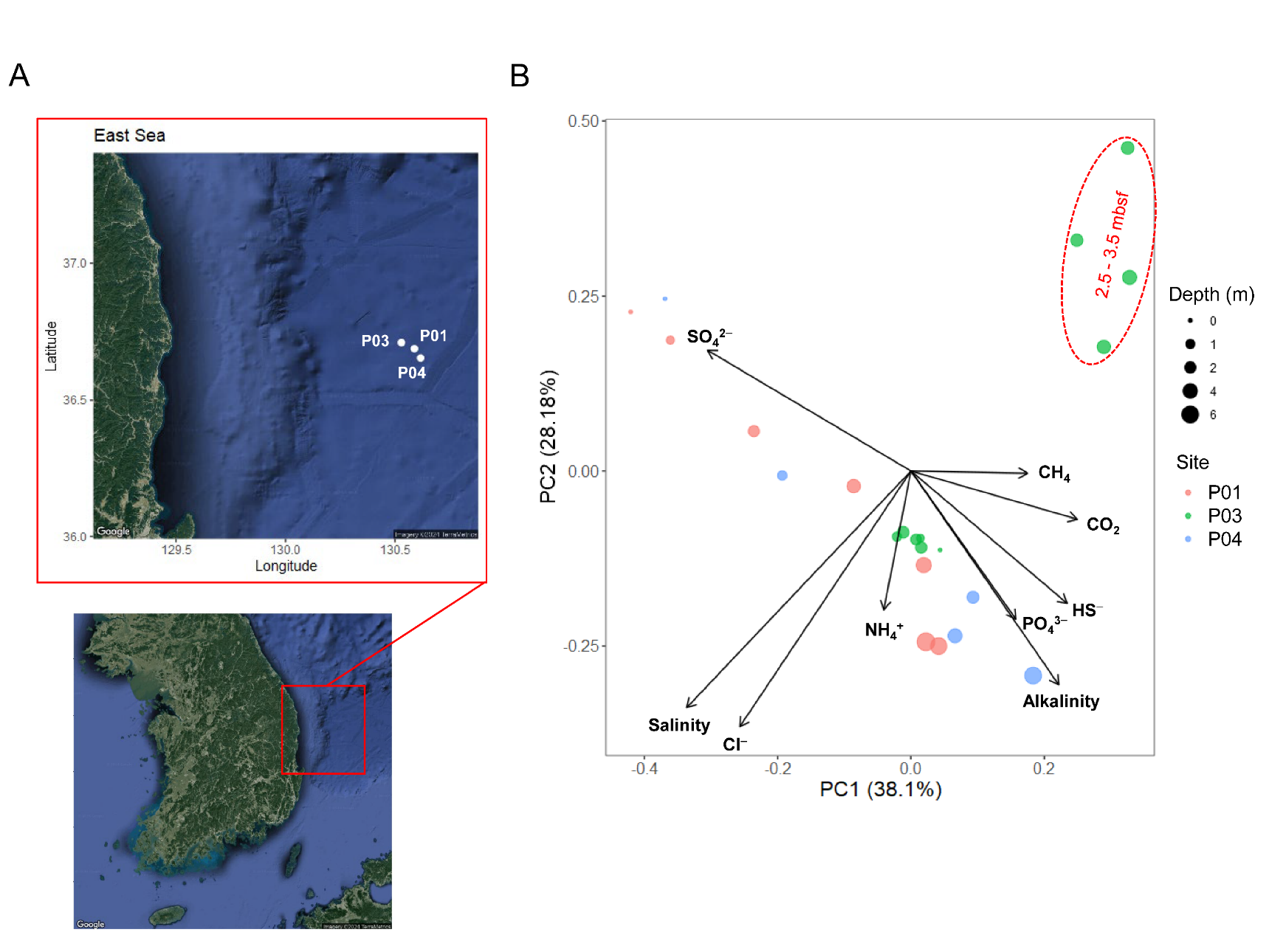

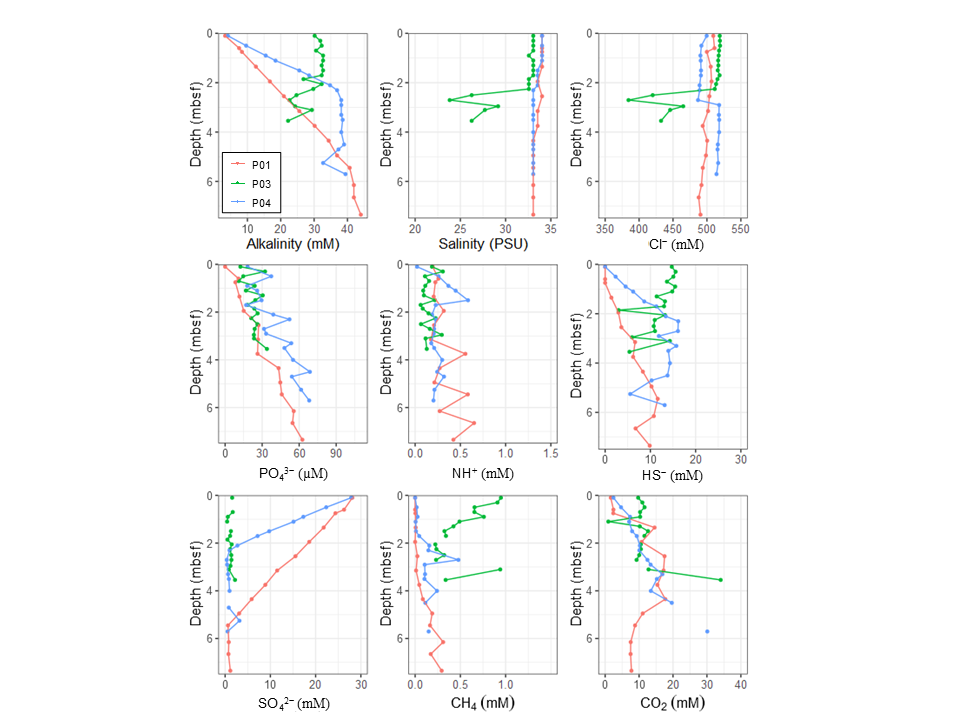


Fig. S1. Depth profiles and principal component analysis (PCA) of porewater geochemical properties from sediment cores (Sites P01, P03, and P04). Depth is shown in meters below seafloor (mbsf). Depth profiles are color-coded by site: P01 (reddish pink), P03 (green), and P04 (sky blue), with the same color scheme applied in the PCA plot. In the PCA plot, the red circle marks samples that were separated as an outlier group, highlighting their distinct geochemical characteristics compared to the other sediments. The PCA was conducted to visualize environmental heterogeneity among the cores, and the variables included represent distinct geochemical properties.


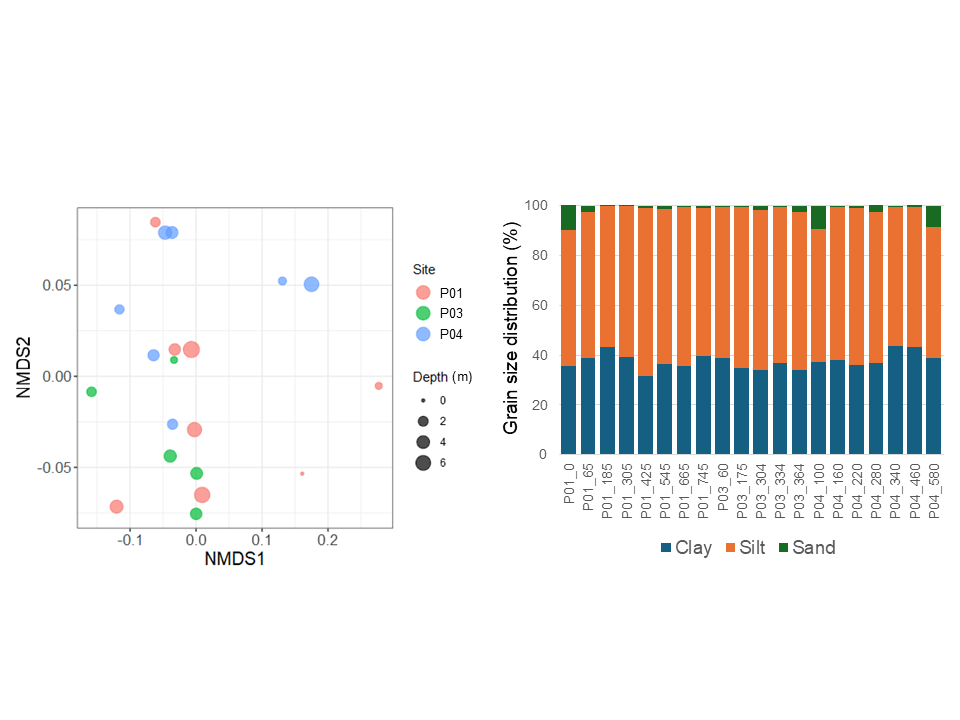


Fig. S2. Measurement of grain size in sediment samples. The NMDS patterns generated using the mode values (i.e., the most frequently occurring grain size within each sample). Distribution (%) of clay, silt, and sand particles in sediments. Numbers shown on sample labels correspond to sample IDs, indicating the combination of site and depth for each sediment sample. A significant difference of sediment samples among the sites (P01, P03, and P04) on the NMDS was determined by PERMANOVA (*P* > 0.05).

A


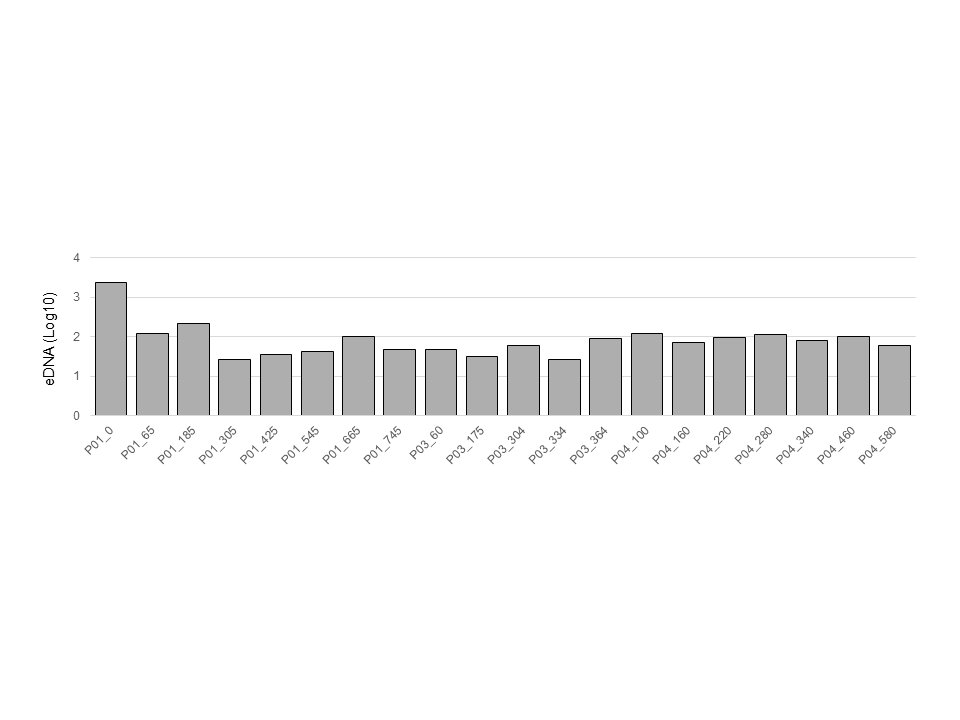


B


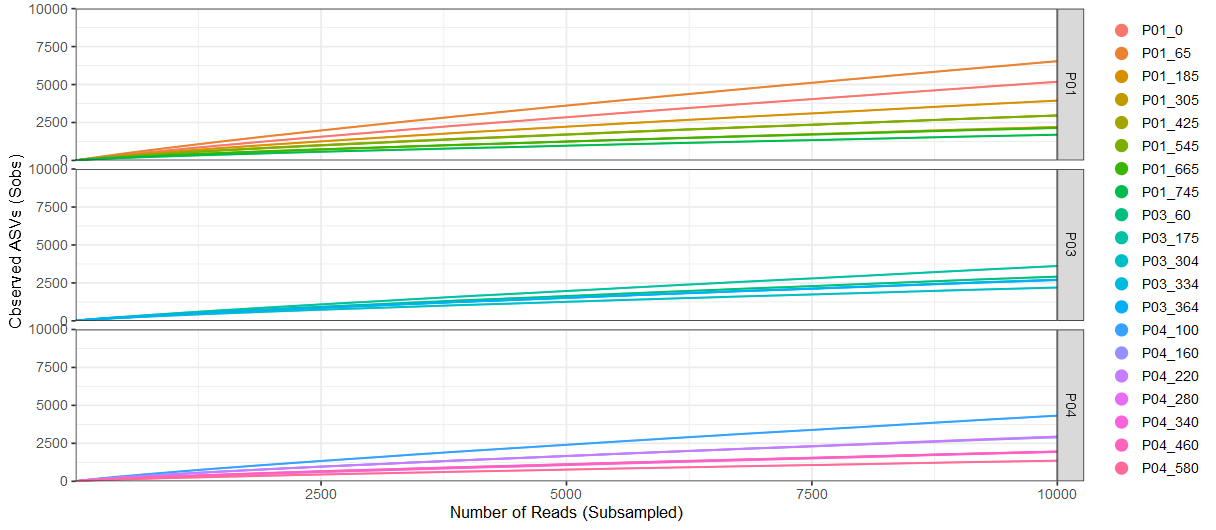


Fig. S3. Concentration of eDNAs and sequencing coverage validation. (A) Concentrations of eDNA in sediment samples. Values are shown as log10-transformed concentrations; as a result of the transformation, no physical units are retained. The original measurements were expressed as nanograms per gram of sediment (ng g⁻¹ sediment). Numbers shown on sample labels correspond to sample IDs, indicating the combination of site and depth for each sediment sample. (B) Rarefaction curves showing observed ASV richness (Sobs) as a function of sequencing depth for all samples. Most curves approached asymptotic trends at approximately 7,000–10,000 reads, indicating sufficient sequencing coverage across all sites and depths. A few surface and mid-depth samples (P01_0 and P01_65) showed slightly increasing trends at the highest read numbers, consistent with locally elevated eDNA concentrations observed in panel (A).


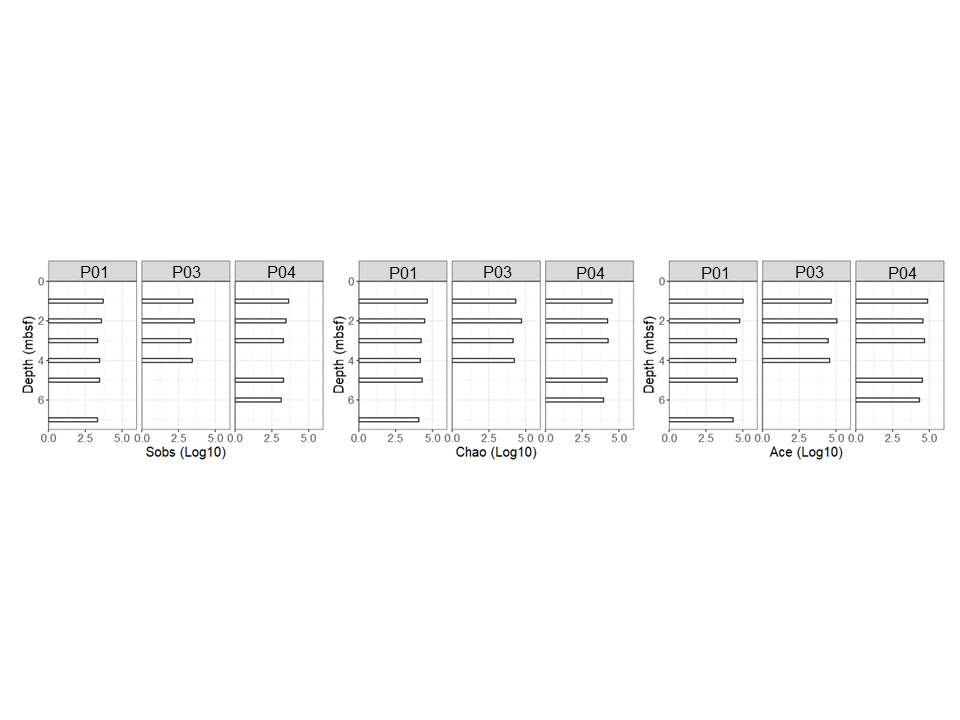


Fig. S4. Microbial diversity. Alpha diversity pattern with Chao and Ace indexes. Pearson correlation analysis confirmed significant negative correlations between diversity indices and depth at P01 (Chao: -0.89, *P* < 0.05; Ace: -0.89, *P* < 0.05) and P04 (Chao: -0.82, *P* < 0.05; Ace: -0.77, *P* < 0.05), whereas correlations at P03 were weak and not significant (Chao: -0.41, *P* > 0.05; Ace: -0.41, *P* > 0.05).


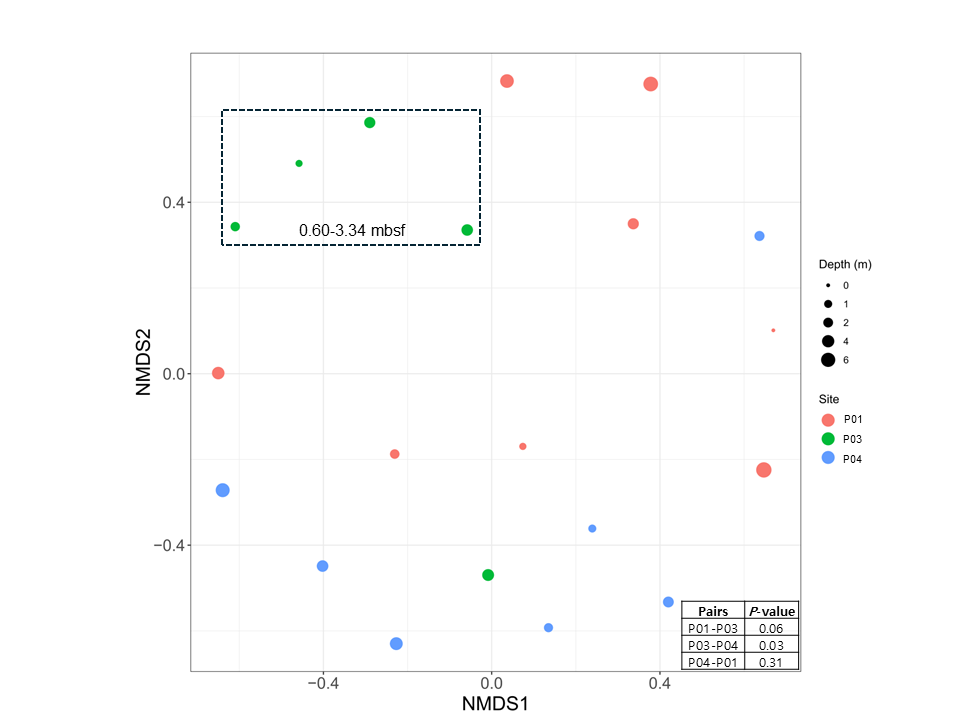


Fig. S5. Microbial beta diversity pattern visualized in NMDS. A significance of differences in microbial communities between each pair of cores was estimated using AMOVA. A dashed ellipse highlights the upper layer of P03 sediments (0.60–3.34 mbsf), which was significantly different from other layers in AMOVA (*P* < 0.05).


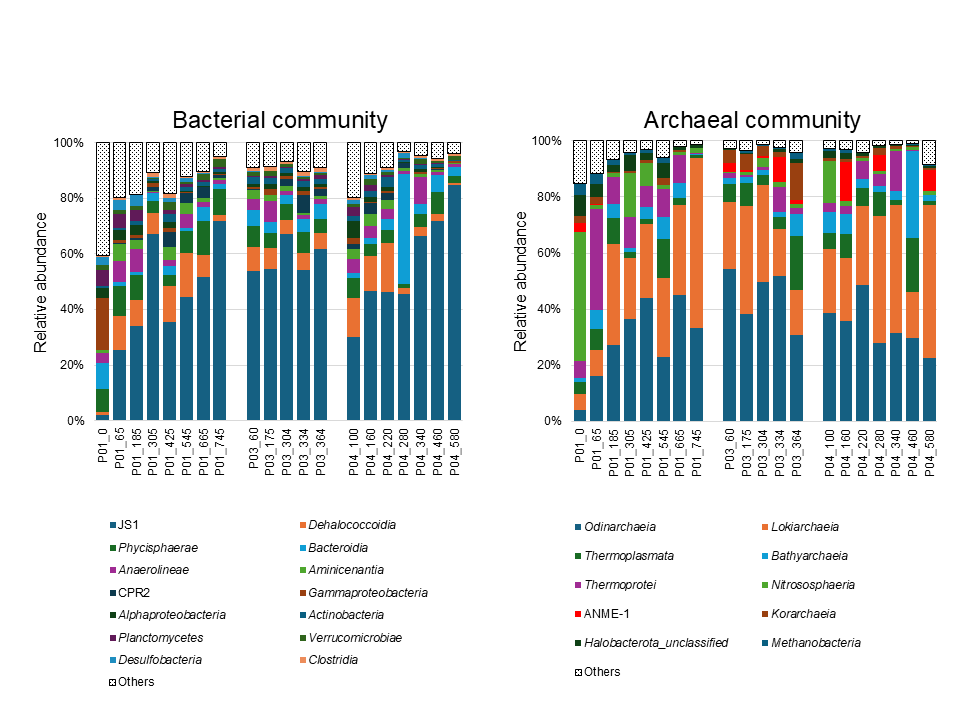


Fig. S6. Microbial community composition at class level. Microbial taxa with a relative abundance of less than 1% within each sample were classified as other groups.


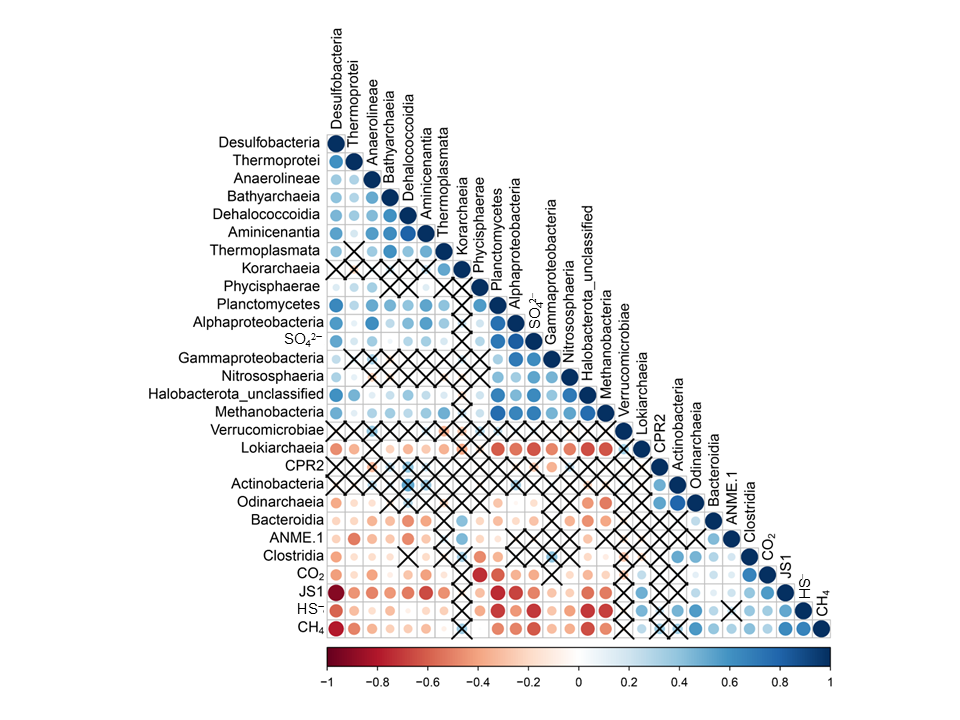


Fig. S7. Correlation of microbial taxa (class level) with HS^−^, SO_4_^2−^, CO_2_, and CH_4_. Correlation coefficients are colored according to the value scale. Positive correlations are displayed in a blue scale while negative correlations are displayed in a red scale. Coefficients not statistically significant (*P* > 0.01) are marked with X.

Table S1. Site information of the 17GH expedition

| Site | Latitude | Longitude | Water Depth | Total Core Length |
| --- | --- | --- | --- | --- |
|  | (^o^N) | (^o^E) | (m) | (m) |
| 17GH-P01 | 36°41′18.147″ | 130°35′17.694″ | 2041 | 7.45 |
| 17GH-P03 | 36°39′14.881″ | 130°36′58.961″ | 2018 | 3.65 |
| 17GH-P04 | 36°42′39.604″ | 130°31′44.786″ | 2032 | 5.81 |

Table S2. Measured values of the dissociated gas hydrate in Site P03 (mbsf, meters below seafloor; psu, practical salinity unit; Cl⁻, chloride; SO_4_^2-^, sulfate; NH_4_^+^, ammonium; PO_4_^3-^, phosphate; δ^18^O, oxygen isotope ratio; δD, hydrogen isotope ratio; δ^13^C_CH4_, carbon isotopic composition of methane; δD_CH4_, hydrogen isotopic composition of methane; VPDB, Vienna Pee Dee Belemnite; V-SMOW, Vienna Standard Mean Ocean Water). The symbol “―” indicates that values were measured but were below the detection limit of analysis.

| Depth (mbsf) | Salinity (PSU) | Cl^⁻^  (mM) | SO_4_^2-^  (mM) | Alkalinity (mM) | NH_4_^+^ (mM) | PO_4_^3-^  (µM) | δ^18^O (‰ V-SMOW) | δD (‰ V-SMOW) | δ^13^C_CH4_ (‰ V-PDB) | δD_CH4_ (‰ V-SMOW) |
| --- | --- | --- | --- | --- | --- | --- | --- | --- | --- | --- |
| 3.21 | 2.43 | 40.07 | ― | 2.06 | 0.23 | 10.29 | 2.17 | 15.50 | ― | ― |
| 3.60 | 6.32 | 100.18 | 0.32 | 6.68 | 0.06 | 11.28 | 1.88 | 16.00 | ― | ― |
| 3.65 | ― | ― | ― | ― | ― | ― | ― | ― | -66.5 | -200.0 |

Table S3. The isotopic composition of headspace gas samples (mbsf, meters below seafloor; δ^13^C_CH4_, carbon isotopic composition of methane; δD_CH4_, hydrogen isotopic composition of methane; δ^13^C_CO2_, carbon isotopic composition of carbon dioxide; VPDB, Vienna Pee Dee Belemnite; V-SMOW, Vienna Standard Mean Ocean Water). The symbol “―” indicates that isotopic values were measured but were below the detection limit of the isotopic analysis.

| Site | Depth  (mbsf) | δ^13^C_CH4_  (‰ V-PDB) | δD_CH4_  (‰ V-SMOW) | δ^13^C_CO2_  (‰ V-PDB) |
| --- | --- | --- | --- | --- |
| P01 | 0.00 | ― | ― | ― |
|  | 0.65 | ― | ― | ― |
|  | 1.85 | ― | ― | ― |
|  | 3.05 | ― | ― | ― |
|  | 4.25 | -62.0 | ― | -24.3 |
|  | 5.45 | -81.0 | ― | -25.1 |
|  | 6.65 | -83.9 | -187.0 | -23.1 |
|  | 7.45 | -80.7 | ― | -21.7 |
| P03 | 0.60 | -62.8 | ― | -21.2 |
|  | 1.76 | -64.0 | ― | -22.9 |
|  | 3.05 | -64.2 | -188.0 | -22.6 |
|  | 3.35 | -66.9 | -197.0 | -19.7 |
|  | 3.65 | -68.5 | -196.0 | -25.9 |
| P04 | 1.01 | ― | ― | ― |
|  | 1.61 | ― | ― | ― |
|  | 2.21 | -92.0 | ― | -25.0 |
|  | 2.81 | -81.7 | -196.0 | ― |
|  | 3.41 | -78.7 | -196.0 | -19.2 |
|  | 4.61 | -75.8 | -197.0 | -22.7 |
|  | 5.81 | -74.6 | -197.0 | -18.0 |

| **Taxonomy** | | **PO1 (cmbsf)** | | | | | | | | **PO3 (cmbsf)** | | | | | **PO4 (cmbsf)** | | | | | | |
| --- | --- | --- | --- | --- | --- | --- | --- | --- | --- | --- | --- | --- | --- | --- | --- | --- | --- | --- | --- | --- | --- |
| **Domain** | **Phylum;Class;Order;Family** | **0** | **65** | **185** | **305** | **425** | **545** | **665** | **745** | **60** | **175** | **304** | **334** | **364** | **100** | **160** | **220** | **280** | **340** | **460** | **580** |
| Bacteria | Caldatribacteriota;JS1;JS1_or;JS1_fa | 1.9 | 25.4 | 34.2 | 67.0 | 35.7 | 44.6 | 51.8 | 71.6 | 53.8 | 54.7 | 67.0 | 54.3 | 61.8 | 30.0 | 46.5 | 46.2 | 45.4 | 66.3 | 72.0 | 84.7 |
|  | Planctomycetota;Phycisphaerae;MSBL9;SG8-4 | 6.7 | 10.3 | 8.3 | 4.3 | 3.8 | 7.8 | 12.2 | 9.4 | 7.6 | 5.4 | 5.6 | 7.5 | 4.9 | 7.0 | 4.1 | 4.7 | 1.5 | 4.7 | 7.8 | 2.4 |
|  | Chloroflexi;Dehalococcoidia;GIF3;GIF3_fa | 0.7 | 9.2 | 4.0 | 6.1 | 9.5 | 13.2 | 6.5 | 1.4 | 6.5 | 5.2 | 3.6 | 4.2 | 3.8 | 10.4 | 9.8 | 15.9 | 1.6 | 2.3 | 1.7 | 0.7 |
|  | Chloroflexi;Anaerolineae;Anaerolineales ;Anaerolineaceae | 3.0 | 5.2 | 4.6 | 0.3 | 1.0 | 3.1 | 1.4 | 1.1 | 2.5 | 6.6 | 0.6 | 0.8 | 1.1 | 3.3 | 2.5 | 3.2 | 0.6 | 9.6 | 1.1 | 1.1 |
|  | Bacteroidota;Bacteroidia;Bacteroidia_uncl. ;Bacteroidia_uncl. | 0.1 | 0.1 | 0.3 | 0.1 | 0.2 | 0.3 | 3.7 | 0.1 | 2.7 | 0.2 | 0.4 | 0.0 | 2.7 | 0.9 | 0.8 | 1.6 | 25.2 | 0.0 | 2.6 | 1.4 |
|  | Acidobacteriota;Aminicenantia;Aminicenantales  ;Aminicenantales_fa | 0.9 | 6.3 | 3.0 | 0.6 | 4.4 | 3.9 | 1.2 | 0.7 | 3.4 | 2.1 | 1.7 | 0.6 | 1.0 | 3.6 | 4.3 | 3.5 | 1.1 | 0.8 | 0.8 | 0.1 |
|  | Patescibacteria;CPR2;CPR2_or;CPR2_fa | 0.1 | 0.1 | 0.9 | 1.1 | 5.5 | 3.5 | 3.9 | 1.3 | 0.4 | 0.3 | 2.6 | 6.5 | 2.5 | 1.6 | 3.9 | 2.5 | 1.7 | 0.1 | 0.3 | 0.2 |
|  | Chloroflexi;Dehalococcoidia;vadinBA26 ;vadinBA26_fa | 0.2 | 1.8 | 2.0 | 1.5 | 3.4 | 1.9 | 1.1 | 0.7 | 1.7 | 1.8 | 1.1 | 1.2 | 1.5 | 2.8 | 2.2 | 1.7 | 0.7 | 1.1 | 0.7 | 0.2 |
|  | Others | 86.6 | 41.6 | 42.7 | 19.0 | 36.5 | 21.7 | 18.0 | 13.5 | 21.4 | 23.7 | 17.2 | 25.1 | 20.8 | 40.6 | 25.9 | 20.8 | 22.0 | 15.2 | 13.1 | 9.2 |
| Archaea | Asgardarchaeota;Odinarchaeia;Odinarchaeia_or  ;Odinarchaeia_fa | 4.0 | 16.2 | 27.3 | 36.4 | 44.0 | 22.9 | 44.9 | 33.5 | 54.5 | 38.3 | 49.8 | 51.7 | 30.9 | 38.8 | 35.9 | 48.6 | 28.0 | 31.5 | 29.8 | 22.7 |
|  | Asgardarchaeota;Lokiarchaeia;Lokiarchaeia_or  ;Lokiarchaeia_fa | 6.0 | 9.2 | 36.1 | 21.9 | 26.5 | 28.2 | 32.3 | 60.4 | 23.7 | 38.6 | 34.6 | 16.9 | 16.0 | 22.8 | 22.5 | 28.2 | 45.4 | 45.8 | 16.3 | 54.5 |
|  | Thermoplasmatota;Thermoplasmata;SG8-5  ;SG8-5_fa | 3.2 | 6.9 | 9.0 | 2.0 | 1.5 | 13.8 | 2.4 | 1.0 | 6.4 | 7.7 | 3.5 | 3.9 | 18.0 | 5.0 | 8.0 | 6.3 | 8.0 | 1.4 | 18.9 | 1.5 |
|  | Crenarchaeota;Bathyarchaeia;Bathyarchaeia_or  ;Bathyarchaeia_fa | 1.2 | 6.8 | 4.9 | 1.6 | 4.5 | 7.7 | 5.1 | 0.6 | 2.2 | 2.4 | 1.6 | 1.9 | 7.9 | 7.4 | 7.2 | 3.4 | 2.4 | 3.5 | 31.3 | 2.0 |
|  | Crenarchaeota;Nitrososphaeria;Nitrosopumilales  ;Nitrosopumilaceae | 46.0 | 1.6 | 1.5 | 15.6 | 8.1 | 1.6 | 1.0 | 1.7 | 0.6 | 0.9 | 3.2 | 2.1 | 1.5 | 14.7 | 1.9 | 1.1 | 0.9 | 0.7 | 1.2 | 1.4 |
|  | Crenarchaeota;Thermoprotei;Thermoproteales  ;Thermoproteaceae | 3.0 | 26.5 | 2.3 | 3.3 | 5.0 | 6.6 | 4.5 | 0.1 | 0.6 | 0.3 | 0.8 | 3.5 | 1.0 | 1.1 | 0.8 | 3.0 | 1.1 | 3.5 | 0.1 | 0.1 |
|  | Crenarchaeota;Thermoprotei;Thermoprotei_uncl.  ;Thermoprotei_uncl. | 3.2 | 7.6 | 6.9 | 7.4 | 2.2 | 3.0 | 2.1 | 0.0 | 0.7 | 0.4 | 0.3 | 5.1 | 1.0 | 2.1 | 1.8 | 3.0 | 3.0 | 10.5 | 0.0 | 0.0 |
|  | Halobacterota;ANME-1;ANME-1_or;ANME-1_fa | 2.9 | 0.1 | 0.0 | 0.0 | 0.0 | 0.0 | 0.0 | 0.0 | 3.2 | 0.8 | 1.0 | 8.8 | 1.3 | 0.0 | 13.8 | 0.0 | 5.8 | 0.0 | 0.0 | 7.5 |
|  | Korarchaeota;Korarchaeia;Korarchaeales ;Korarchaeaceae | 2.6 | 2.6 | 0.5 | 0.7 | 1.0 | 2.5 | 1.0 | 0.1 | 4.5 | 5.5 | 3.3 | 1.7 | 13.0 | 1.2 | 1.2 | 0.8 | 2.6 | 0.8 | 0.3 | 0.8 |
|  | Halobacterota;Halobacterota_uncl. ;Halobacterota_uncl.;Halobacterota_uncl. | 7.6 | 4.7 | 2.2 | 5.8 | 2.5 | 5.1 | 0.8 | 1.0 | 0.4 | 0.5 | 0.2 | 1.2 | 1.5 | 2.4 | 2.3 | 1.0 | 0.4 | 0.5 | 0.3 | 0.6 |
|  | Others | 20.3 | 17.8 | 9.2 | 5.3 | 4.7 | 8.6 | 6.0 | 1.5 | 3.3 | 4.6 | 1.7 | 3.2 | 7.9 | 4.4 | 4.8 | 4.7 | 2.3 | 1.7 | 1.7 | 9.0 |

Table S4. Relative abundance of microbial taxa at family level. Microbial taxa with a relative abundance of less than 1% within each sample were classified as other groups.

Table S5. List of ASVs selected based on LDA scores.

| **Pairs** | **ASVs** | **Class** | **LDA** | ***P*-value** |
| --- | --- | --- | --- | --- |
| P01-P03-P04 | ASV101 | All | 3.05 | 0.01 |
| P01-P03 | ASV3 | P03 | 4.49 | 0.01 |
| P03-P04 | ASV3 | P03 | 4.47 | 0.01 |
| P01-P03 | ASV12 | P03 | 4.09 | 0.01 |
| P03-P04 | ASV12 | P03 | 4.07 | 0.01 |
| P03-P04 | ASV26 | P03 | 3.80 | 0.03 |
| P01-P03 | ASV32 | P03 | 3.62 | 0.02 |
| P01-P03 | ASV45 | P03 | 3.62 | 0.00 |
| P03-P04 | ASV45 | P03 | 3.53 | 0.01 |
| P01-P03 | ASV50 | P03 | 3.54 | 0.02 |
| P03-P04 | ASV50 | P03 | 3.48 | 0.01 |
| P01-P03 | ASV58 | P03 | 3.52 | 0.02 |
| P03-P04 | ASV58 | P03 | 3.45 | 0.03 |
| P01-P03 | ASV81 | P03 | 3.29 | 0.02 |
| P01-P03 | ASV127 | P03 | 3.15 | 0.02 |
| P03-P04 | ASV127 | P03 | 3.13 | 0.03 |
| P01-P03 | ASV187 | P03 | 2.98 | 0.02 |

**References**

1. Kim KR, Kim K. What is happening in the East Sea (Japan Sea)?: Recent chemical observations during CREAMS 93-96. J Korean Soc Oceanogr. 1996;31(4):164-72.

2. Hyun JH, Mok JS, You OR, Kim D, Choi DL. Variations and controls of sulfate reduction in the continental slope and rise of the Ulleung Basin off the southeast Korean upwelling system in the East Sea. Geomicrobiol J. 2010;27(2):212-22.

3. Kim JH, Torres ME, Choi J, Bahk JJ, Park MH, Hong WL. Inferences on gas transport based on molecular and isotopic signatures of gases at acoustic chimneys and background sites in the Ulleung Basin. Org Geochem. 2012;43:26-38.

4. Haacke R, Hyndman R, Park K, Yoo D, Stoian I, Schmidt U. Migration and venting of deep gases into the ocean through hydrate-choked chimneys offshore Korea. Geology. 2009;37(6):531-4.

5. Horozal S, Lee GH, Bo YY, Yoo DG, Park KP, Lee HY, et al. Seismic indicators of gas hydrate and associated gas in the Ulleung Basin, East Sea (Japan Sea) and implications of heat flows derived from depths of the bottom-simulating reflector. Mar Geol. 2009;258(1-4):126-38.

6. Torres ME, Kim JH, Choi JY, Ryu BJ, Bahk JJ, Riedel M, et al.: Occurrence of high salinity fluids associated with massive near-seafloor gas hydrate deposits. In: Proceedings of the 7th International Conference on Gas Hydrates (ICGH 2011), Edinburgh, Scotland, United Kingdom. 2011.

7. Chun JH, Ryu BJ, Son BK, Kim JH, Lee JY, Bahk JJ, et al. Sediment mounds and other sedimentary features related to hydrate occurrences in a columnar seismic blanking zone of the Ulleung Basin, East Sea, Korea. Mar Pet Geol. 2011;28(10):1787-800.

8. Lee JW, Kwon KK, Azizi A, Oh HM, Kim W, Bahk JJ, et al. Microbial community structures of methane hydrate-bearing sediments in the Ulleung Basin, East Sea of Korea. Mar Pet Geol. 2013;47:136-46.

9. Choi J, Yi B-Y, Kim Y, Jeong E-J, Um I-K. Spatial variations in organic matter sources and biogeochemical processes in the East Sea. Front Mar Sci. 2025;12:1571191.

10. Jang G, Hong S, Oh J, Kim Y-I, Kim M, Lee H. Statistical analysis of the association between El Niño and the biological carbon pump in the East Sea (Japan Sea). Sci Rep. 2024;14(1):26582.

11. Kim D, Choi M-S, Oh H-Y, Song Y-H, Noh J-H, Kim KH. Seasonal export fluxes of particulate organic carbon from 234 Th/238 U disequilibrium measurements in the Ulleung Basin 1 (Tsushima Basin) of the East Sea 1 (Sea of Japan). J Oceanogr. 2011;67:577-88.

12. Kim J-H, Park M-H, Ryu J-S, Jang K, Choi J, Park S, et al. Exploring the pore fluid origin and methane-derived authigenic carbonate properties in response to changes in the methane flux at the southern Ulleung Basin, South Korea. Front Mar Sci. 2024;10:1156918.
